# Cortical haemodynamic responses predict individual ability to recognise vocal emotions with uninformative pitch cues but do not distinguish different emotions

**DOI:** 10.1002/hbm.26305

**Published:** 2023-05-10

**Authors:** Ryssa Moffat, Deniz Başkent, Robert Luke, David McAlpine, Lindsey Van Yper

**Affiliations:** ^1^ School of Psychological Sciences Macquarie University Sydney New South Wales Australia; ^2^ International Doctorate of Experimental Approaches to Language and Brain (IDEALAB) Universities of Potsdam, Germany; Groningen, Netherlands; Newcastle University, UK; and Macquarie University Australia; ^3^ Department of Otorhinolaryngology/Head and Neck Surgery, University Medical Center Groningen University of Groningen Groningen The Netherlands; ^4^ Research School of Behavioral and Cognitive Neuroscience, Graduate School of Medical Sciences University of Groningen Groningen The Netherlands; ^5^ Macquarie University Hearing, and Department of Linguistics Macquarie University Sydney New South Wales Australia; ^6^ Bionics Institute East Melbourne Victoria Australia; ^7^ Institute of Clinical Research University of Southern Denmark Odense Denmark

**Keywords:** affect, emotion recognition, fNIRS, prosody

## Abstract

We investigated the cortical representation of emotional prosody in normal‐hearing listeners using functional near‐infrared spectroscopy (fNIRS) and behavioural assessments. Consistent with previous reports, listeners relied most heavily on F0 cues when recognizing emotion cues; performance was relatively poor—and highly variable between listeners—when only intensity and speech‐rate cues were available. Using fNIRS to image cortical activity to speech utterances containing natural and reduced prosodic cues, we found right superior temporal gyrus (STG) to be most sensitive to emotional prosody, but no emotion‐specific cortical activations, suggesting that while fNIRS might be suited to investigating cortical mechanisms supporting speech processing it is less suited to investigating cortical haemodynamic responses to individual vocal emotions. Manipulating emotional speech to render F0 cues less informative, we found the amplitude of the haemodynamic response in right STG to be significantly correlated with listeners' abilities to recognise vocal emotions with uninformative F0 cues. Specifically, listeners more able to assign emotions to speech with degraded F0 cues showed lower haemodynamic responses to these degraded signals. This suggests a potential objective measure of behavioural sensitivity to vocal emotions that might benefit neurodiverse populations less sensitive to emotional prosody or hearing‐impaired listeners, many of whom rely on listening technologies such as hearing aids and cochlear implants—neither of which restore, and often further degrade, the F0 cues essential to parsing emotional prosody conveyed in speech.

## Graphical abstract

We investigate the impact of attenuating fundamental frequency, intensity, and speech rate cues on vocal emotions using novel speech stimuli, measures of accuracy, and functional near‐infrared spectroscopy (fNIRS). Combining three assessments, we demonstrate: (1) listeners' accuracy in recognising emotions decreases most when fundamental frequency cues are attenuated, (2) fNIRS is not suited to distinguishing between cortical representations of individual emotions conveyed in natural speech, and (3) fNIRS is sensitive to increased cortical activity in right superior temporal gyrus in listeners who struggle most to retrieve emotional meaning from speech with attenuated fundamental frequency cues.

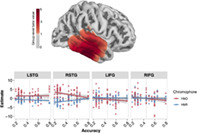

## 1 INTRODUCTION

Recognising emotions in speech is important to successful communication, social interaction, and quality of life (Lindner & Rosén, 2006; Luo et al., 2018; Zinchenko et al., 2018). In face‐to‐face conversation, both visual and auditory cues convey information about an interlocuter's emotional state. However, in many common listening situations, such as during a telephone conversation, when a talker is occluded from view, or in a poorly lit room, visual information may be absent or limited, and listeners may rely entirely on vocal information to decode their interlocuter's emotional state. In these circumstances, degraded vocal information—or reduced capacity to process it—can impair communication and strain social interaction. The main acoustic features conveying vocal emotions are the fundamental frequency, or F0, which corresponds to the rate of vibration of the vocal folds and is the acoustic correlate of voice pitch; the intensity (sound pressure level) at which speech is uttered and the acoustic correlate of loudness; and speech rate, the number of syllables spoken per second, corresponding to the acoustic quality of tempo (Frick, 1985; Juslin & Laukka, 2001; Scherer et al., 2003). Variations in these acoustic features over time provide listeners cues to emotional meaning in vocalisations, while any reduction in the variations in these features attenuates the cues with which listeners recognise vocal emotions, rendering the cues to the emotional meaning of the speech less informative (e.g., Metcalfe, 2017; Scherer et al., 2003).

Emotions in speech are conveyed through prosody, comprising melodic and rhythmic cues such as F0, intensity, and speech rate (Scherer et al., 2003). Listeners rely most heavily on variations over time in F0 cues (Banse & Scherer, 1996; Metcalfe, 2017; Patel et al., 2011; Scherer et al., 2003), but the degree to which they can exploit variations in intensity and speech rate in the absence of F0 information remains unclear. However, listeners appear to weight acoustic features according to the usefulness of the conveyed cues, such that when attenuated variations in an acoustic feature render cues uninformative, listeners rely more on other acoustic features with more informative cues (“weighting‐by‐reliability”; Toscano & McMurray, 2010). With F0 playing a primary role as a stand‐alone acoustic feature conveying cues to vocal emotions, normal‐hearing listeners recognise vocal emotions with variations preserved only in F0, but less so when only intensity or speech‐rate cues are preserved (Metcalfe, 2017). Hearing‐impaired listeners, including those who use listening devices such as hearing aids (HAs) or cochlear implants (CIs), show difficulties identifying emotions in speech (Chatterjee et al., 2015; Everhardt et al., 2020; Goy et al., 2018; Most & Aviner, 2009), likely the result of reduced spectral resolution (wider auditory filters or channels interactions in implants)—especially for listeners with severe‐to‐profound hearing loss—and a compressed intensity range in the impaired inner ear (loudness recruitment). No current hearing device is able to entirely compensate for these impairments due to the underlying physiological limitations of hearing loss. The hypothesis that acoustic cues to emotional prosody are perceptually weighted by their reliability (Toscano & McMurray, 2010) suggests that hearing‐impaired listeners, faced with relatively weak F0 cues, might make use of cues conveyed by more intact acoustic features.

Neuroimaging studies using functional magnetic resonance imaging (fMRI) or functional near‐infrared spectroscopy (fNIRS) indicate that speech with uninformative F0 cues most commonly evokes reduced activity, relative to natural speech, in bilateral superior temporal gyrus (STG; Lawrence et al., 2018; Pollonini et al., 2014; Wild et al., 2012). Nonetheless, Evans et al. (2014) and Wijayasiri et al. (2017) reported no difference in bilateral STG, and Meyer et al. (2004) reported increased activity in right STG when F0 cues were uninformative. Elevated cognitive effort in processing speech with reduced emotional cues is also associated with elevated activity in left inferior frontal gyrus (IFG; Evans et al., 2014; Lawrence et al., 2018; Wijayasiri et al., 2017; Wild et al., 2012). Nevertheless, consistent with the proposed specialisation of right STG for decoding changes in pitch (Flinker et al., 2019; Friederici & Gierhan, 2013; Poeppel, 2003), haemodynamic activity measured using fMRI and fNIRS evoked by recognisable emotions relative to neutral or unemotional speech, is greater in right, compared to left, STG (Beaucousin et al., 2007; Witteman et al., 2012; Zhang et al., 2018; Zhen et al., 2021). Further, poor recognition of vocal emotions is associated with right‐lateralisation of cortical activity measured with electroencephalography (EEG; Cartocci et al., 2021; Kislova & Rusalova, 2009), and may reflect increased attention to the emotional content of the speech (Kislova & Rusalova, 2009), or in the case of hearing‐impaired listeners, neuroplastic changes resulting from prolonged deafness (Cartocci et al., 2021).

Here, we assess the ability of normal‐hearing listeners to exploit secondary cues (intensity and speech rate) when F0 cues are uninformative for emotional prosody. Attenuating variations in the relevant acoustic cues of English sentences spoken by a native Australian‐English talker, we use a four‐alternative, forced‐choice recognition task to assess recognition of vocal emotions, confirming that listeners' ability to recognise emotions in speech depends on the availability of F0 cues. Then, using fNIRS with dense optode coverage and short channels to exclude extracerebral and other signal components such as Mayer waves, we find that emotion‐specific confounds are unlikely to arise in fNIRS measurements of emotional speech, as similar cortical signatures are observed for any investigated emotion. Finally, assessing cortical haemodynamic activity evoked by vocal emotions in natural speech and in speech manipulated to render F0 cues uninformative, we demonstrate that the amplitudes of haemodynamic activity in right STG are predictive of listeners' abilities to extract emotional meaning from speech in which F0 cues to vocal emotions are rendered uninformative. Our data suggest that haemodynamic responses from the right STG activity represent a possible objective measure of the recognition of vocal emotions when F0 cues are attenuated.

## 2 METHODS

### 2.1 Participants

For all assessments, participants were recruited from Macquarie University (largely students as well as several staff members). Participants were aged 18–36 years old, right‐handed, had no known neurological or psychological disorders, and presented with normal hearing, that is, pure‐tone audiometric thresholds indicating no more than slight hearing losses, as defined by the American Speech Language Hearing Association (≤25 dB HL for all octave frequencies between 250 and 8000 Hz; Clark, 1981). The first behavioural emotion recognition assessment included 40 participants (20 female, 20 male; *M* age = 24 years; *SD* = 6 years). Then, 21 participants were recruited for each of 2 fNIRS sessions: 17 participants completed both sessions, 4 more completed the first session but not the second, and 4 others completed the second session but not the first (emotions in natural speech: 10 females, 11 males, *M* age = 27.4 years, *SD* = 4.7 years; emotions with and without attenuated F0 cues: 9 females, 12 males, *M* age = 27 years, *SD* = 4.6 years). Ethical approval for all sessions was obtained from Macquarie University Human Research Ethics Committee (ID 5297). Written informed consent was collected from all participants, who received a participant fee for their involvement.

### 2.2 Stimuli

Stimuli for all assessments comprised six‐syllable sentences with rhyming pseudo‐words and real function‐words, such as “*the ziffox is dorval*” and *“the miffox is lorval”* (see Supplementary Material S1 for full list). The rhyming pseudo‐words alleviate semantic confounds and minimise phonetic differences (e.g., vowel length and formants) while preserving English phonetic sequences, and the key words preserved English sentence structure. This combination effectively isolates the acoustic signal as the sole carrier of information about vocal emotion (Banse & Scherer, 1996; Pell & Kotz, 2011). Acoustic recordings were obtained in an anechoic chamber using a Røde NT1 microphone with a pop cover (RØDE Microphones, Silverwater, Australia) connected to an RME Fireface UC sound card (Audio AG, Haimhausen, Germany). The sentences were digitally recorded using Adobe Audition (version 13.0.8.43; Adobe Systems, Mountain View, USA) on a personal computer at a sampling rate of 48 kHz (32 bits mono). The 27‐year‐old female native speaker of Australian English pronounced the sentences employing the prosodies *angry*, *happy*, *sad*, and *unemotional* (acoustic features described in Table 1).

**TABLE 1:** Mean and standard deviation of each acoustic feature for recordings of each emotion. dB is relative to the maximum measured RMS.

Emotion	F0 (Hz)			Intensity (dB)		Speech rate (syl/s)	
	*M*	*SD*	Range	*M*	*SD*	*M*	*SD*
*Angry*	241.47	6.14	100.07–349.49	−0.99	1.01	4.50	0.07
*Happy*	243.31	10.29	84.06–355.10	−10.97	0.85	5.57	0.18
*Sad*	215.97	11.44	76.50–327.76	−17.76	1.44	4.70	0.31
*Unemotional*	167.04	8.37	78.28–237.14	−15.04	0.58	4.68	0.26
All speech	216.95	32.70	76.50–355.10	−11.19	6.59	4.86	0.47

**FIGURE 1:**
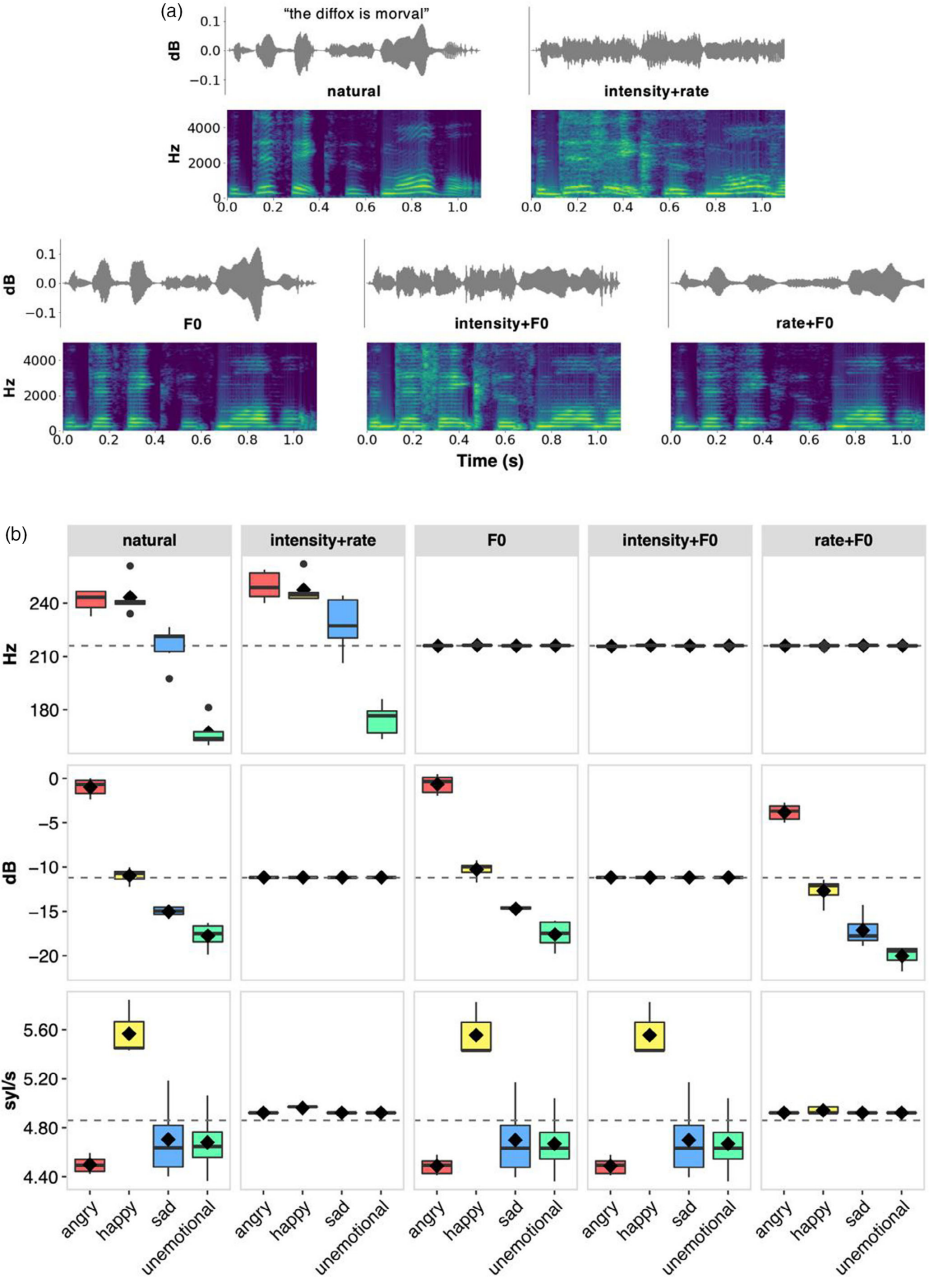
(a) Example waveforms and narrowband spectrograms per condition, here showing the recording of “the diffox is morval” in *happy* prosody. (b) Measured acoustic properties for each emotion within each experimental condition. Where variations in a feature were not attenuated, the distribution of the emotions resembles the *natural* condition. Where variations in a feature have been attenuated, all emotions have the target mean value. Solid bar indicates median, diamond indicates mean, the box includes inter‐quartile range (IQR), whiskers indicate values within 1.5 times the IQR above the 75th or below the 25th percentile, dots indicate values outside 1.5 times the IQR. Dashed line average across all emotions, that is, target value.

Variations in the relevant acoustic features along the sentence‐length trajectory were attenuated, rendering uninformative the associated acoustic cues to emotional prosody. The recordings were manipulated to create five conditions, named for the attenuated acoustic features: *natural*, *intensity* + *rate*, *F0*, *intensity* + *F0*, and *rate* + *F0* (Figure 1). This was achieved by manipulating the acoustic signal for each sentence so that variations in the relevant acoustic feature were equalised to the mean value across all stimulus sentences. The mean F0 was 216.95 Hz (*SD =* 32.70 Hz), while the mean intensity calculated in dB relative to the maximum RMS was −11.19 (*SD* = 6.59), and the mean speech rate was 4.86 syl/s (*SD* = 0.47 syllables/s). Variations in F0 were attenuated to 217 Hz (rounded up from the overall mean of 216.95 Hz) using the PyWORLD Python package (version 0.2.11; Morise et al., 2016). Variations in intensity were attenuated to −11.19 dB relative to max RMS using Praat software (version 6.1.16; Boersma & Weenink, 2018). Sentence‐level variation in speech rate was scaled to 4.82 syllables/s using the Python package AudioTSM (version 0.1.2; Muges, 2017). Where variations were attenuated in more than one acoustic feature simultaneously, variations in F0 were first attenuated, then speech rate, followed by intensity, with overall differences in that acoustic feature kept intact (i.e., F0 in *natural* and *intensity* + *rate* conditions; intensity in *natural*, *F0*, *rate* + *F0* conditions; speech rate in *natural*, *F0*, *intensity* + *F0* conditions). Further details on stimulus generation are provided in S1 of Supplementary Material.

In the first behavioural assessment of emotion recognition, the individual sentences for each *angry*, *happy*, *sad*, and *unemotional* were presented in the five conditions: *natural*, *intensity* + *rate*, *F0*, *intensity* + *F0*, and *rate* + *F0*. Recognition was assessed in a new set of listeners with the four emotions presented only in the *natural* and *F0* conditions, before and after the second fNIRS session.

Changes in cortical oxygenation were measured using fNIRS and a block‐design paradigm. For the first fNIRS session, five sentences conveying each emotion in natural speech were concatenated, yielding ~7.25 s blocks of *angry*, *happy*, *sad*, and *unemotional* natural speech. For the second fNIRS session, the five sentences conveying *happy* and *sad* in each natural speech and speech with attenuated F0 variations were concatenated yielding ~7.25 blocks (*happy*, *sad*, *F0_happy*, *F0_sad*). Stimuli for both fNIRS sessions also included *control* stimuli consisting of 7.25 s of silence and attention‐keeping stimuli consisting of the above‐named blocks with a 500‐ms 400‐Hz tone overlayed at a random time point, which were used to elicit button‐presses confirming participants were attentive. Code for generating stimuli, and stimulus files, are archived at https://osf.io/352qc/.

### 2.3 Procedure

The behavioural and fNIRS assessments took place in a sound‐treated booth, with the lights and computer screens turned off during fNIRS recordings to minimise ambient light. In all assessments, auditory stimuli were presented diotically (i.e., identical at the two ears) in a randomised order using Presentation software (version 20.2; NeuroBehavioral Systems Inc., 2020) via an RME Fireface UC (Audio AG).

#### 2.3.1 Behavioural assessment of emotion recognition (Experiment 1)

Participants completed a four‐alternative forced‐choice (4‐AFC) emotion recognition assessment. The assessment consisted of four practise trials (*angry*, *happy*, *sad*, *unemotional* in natural speech, each presented once) with/out feedback, followed 100 test trials (all 5 sentence‐length stimuli for each 4 emotions and 5 conditions were presented once). Participants were seated in front of a desktop computer. For each trial, participants viewed a screen with the question “Which emotion did you hear?” and the alternatives *unemotional*, *happy*, *sad*, and *angry*. Participants responded using the top four buttons on an RB‐840 button box (Cedrus Corporation, San Pedro, USA) and no feedback on the accuracy was presented to the participant. Stimuli were presented through Etymotic Research ER‐2 insert earphones (Etymotic Research, Inc.) at a mean level of 60 dBA. The same procedure was used for subsequent behavioural assessments in which only *natural* and *F0* conditions were presented, and participants completed the assessment once before and once after the fNIRS recording with stimuli were presented at a mean level of 60 dBA in first assessment and 70 dBA for second assessment. (Stimulus level was increased to ensure a reliable signal‐to‐noise ratio during fNIRS recordings (Weder et al., 2018). Sound‐level calibration described in Supplementary Material S1).

#### 2.3.2 fNIRS listening task (Experiments 2 and 3)

To measure cortical brain activity evoked by vocal emotions in natural speech, fNIRS recordings were made while participants listened to natural speech with different emotional content (Experiment 2: *angry*, *happy*, *sad*, *unemotional*) and to speech (Experiment 3: *happy and sad*) both natural and with attenuated F0 cues. In both cases, participants were instructed that they would hear both speech and silence, and that their task was to remain still throughout the recording and listen to the speech and silence. They were also informed that they occasionally would hear a tone overlapping with the speech and that they should indicate they heard the tone by pressing the top left button on the button box with their left index finger. The task began with four familiarisation trials to familiarise participants with the pseudo‐sentences and the button‐press task. Subsequently, the main part of the task commenced: experimental ~7.25 s blocks of speech (*angry*, *happy*, *sad*, *unemotional*, or *happy*, *sad*, *F0_happy*, F0_*sad*) and silence (control) were each presented 20 times in a randomised order (*n* = 100). In each case, the five stimuli containing the pure tone were each presented twice across the task (*n* = 10). In total, each session consisted of 114 trials separated by inter‐stimulus intervals of random lengths in the range 13–23 s (Figure 3a). The total duration of fNIRS recordings was ~50 min. Stimuli were presented through Etymotic Research ER‐3 insert earphones (Etymotic Research, Inc.) at a mean level of 70 dBA.

### 2.4 fNIRS equipment and optode positions

fNIRS recordings were made with a NIRScoutX (NIRx Medical Technologies LLC) with 24 LED sources and 32 avalanche photodiode detectors. The sources emitted wavelengths of 760 and 850 nm with a sampling rate of 2.6 Hz. The optodes were mounted onto mesh caps marked with International 10/10 positions (Easycap GmbH) using grommets and spacers to maintain a maximum 30‐mm separation (NIRx Medical Technologies LLC).

A montage of 24 sources, 16 detectors, and 8 short detectors was used to record from bilateral STG, IFG, and MFG (Figure 3b,c). To cover these brain areas, our montage comprised 60 long channels (source‐detector pairs ~30 mm apart), along with 8 short channels (source‐detector pairs 8 mm apart), one in each right and left IFG and MFG, and two in each right and left STG to account for location‐dependent heterogeneity in the extracerebral signals (Brigadoi & Cooper, 2015; Gagnon et al., 2012; Y. Zhang et al., 2015). Optode positions were determined using the AAL2 atlas in the fOLD toolbox (Rolls et al., 2015; Tzourio‐Mazoyer et al., 2002; Zimeo Morais et al., 2018), by selecting all suggested channels from the 10/10 and 10/5 systems over bilateral STG, IFG, and MFG (Shader et al., 2021).

### 2.5 Data analysis

#### 2.5.1 Behavioural emotion recognition in speech with and without attenuated cues

In the first behavioural assessment (Experiment 1), the 4‐AFC data were used to evaluate listeners' accuracy in recognising vocal emotions for each manipulation, and for exploring differences in accuracy between consecutive conditions, that is, in the order (a) *natural*, (b) *intensity* + *rate*, (c) *F0*, (d) *intensity* + *F0*, and (e) *rate* + *F0*. Additionally, confusion matrices were constructed using the response rate for each alternative for each presented emotion in each condition to obtain confusion rates.

Accuracy data (4000 trials, 2606 correct responses) were used to fit generalised linear mixed‐effects (GLMMs) models with a logit link function using the lme4 package (version 1.1.21; Bates, Mächler, et al., 2015) in the R language (version 3.6.3; R Core Team, 2020) within the Rstudio IDE (version 1.3.959; Rstudio Team, 2020). To predict the difference in accuracy between consecutive conditions, four sliding difference contrasts (Schad et al., 2020) were defined between consecutive conditions (*natural* vs. *rate* + *intensity*, *rate* + *intensity* vs. *F0*, *F0* vs. *intensity* + *F0*, and *intensity* + *F0* vs. *rate* + *F0*). Each contrast itself constituted a hypothesis (Schad et al., 2020), and was included as a fixed effect in the model predicting overall accuracy (all investigated emotions together) and four additional models predicting accuracy for each emotion separately. Models were simplified following the approach of Bates, Kliegl, et al. (2015) and Bates, Mächler, et al. (2015), by first fitting the maximal model comprising the four contrasts as fixed effects, with uncorrelated random slopes and intercepts of each contrast per participant, sentence, and in the model assessing listeners' overall accuracy, Emotion. Next, random‐effects terms explaining very little variance (*SD* < 0.01) were removed. Subsequently, a likelihood ratio test was performed for each remaining random‐effect term to confirm that the model explained a significantly larger proportion of variance with that term. Unstandardised coefficients are reported for each model, as all predictor variables input into the model are in the same units (Supplementary Table S2a). Additionally, goodness‐of‐fit of the final GLMM is reported with the marginal *R* ^2^, the variance accounted for by fixed effects, and conditional *R* ^2^, the variance accounted for by both fixed and random effects, as a tandem *R* ^2^_*m*/*c*_ calculated using the performance R package (version 0.8.0; Lüdecke et al., 2021) in Supplementary Table S2a.

Before and after the second fNIRS session (Experiment 3), listeners' accuracy in recognising vocal emotions (*angry*, *happy*, *sad*, *unemotional*) conveyed in natural speech and speech with uninformative F0 cues was assessed. The data (1680 trials, 1177 correct responses) were used to fit GLMMs with a logit function following the model‐building approach described above. To test the a priori hypotheses of lower accuracy in recognising vocal emotions conveyed in speech with attenuated variations in F0 relative to emotions in natural speech, and increased accuracy with increasing exposure to the stimuli, treatment contrasts were defined between (a) natural speech and speech with attenuated variations in F0, and (b) accuracy before and after the fNIRS recording.

#### 2.5.2 fNIRS haemodynamic response amplitude evoked by vocal emotions

Quantitative analyses of haemodynamic response amplitude (Huppert, 2016) were undertaken with waveform figures generated for visual inspection (Figure S3b in Supplementary Material S3 and Figure 5; generation procedure detailed in Supplementary Material S3). All analyses were performed using MNE (version 0.23; Gramfort et al., 2013), MNE‐NIRS (version 0.1.2; Luke, Larson, et al., 2021), and NiLearn (version 0.70; Abraham et al., 2014). For each fNIRS session, only experimental‐conditions trials were analysed (Session 1: *angry*, *happy*, *sad*, *unemotional*, *control*, Session 2: *happy*, *sad*, *F0_happy*, *F0_sad*, *control*). Familiarisation and attention trials, as well as trials in which a button press was made whether intentionally or mistakenly, were excluded from the analysis.

##### First level

The generalised linear model (GLM) approach was taken to quantify the amplitude of evoked haemodynamic responses measured in each fNIRS session (Experiments 2 and 3). The sampling rate of the recorded signal was reduced from 2.6 to 0.6 Hz (Luke, Larson, et al., 2021; Luke, Shader & McAlpine, 2021) as the scalp‐coupling index (SCI; Pollonini et al., 2014) was not calculated, alleviating the need for higher frequencies. The signal was converted from raw intensity to optical density, using absolute raw intensity values. Next, the signal was converted to concentrations of oxygenated (HbO) and deoxygenated (HbR) haemoglobin using the Modified Beer–Lambert law (Delpy et al., 1988; Kocsis et al., 2006) with a partial pathlength factor of 0.1. Of note, the partial pathlength accounts for both differential pathlength factor (DPF) and partial volume correction (PVC), where (DPF = 6)/(PVC = 60) is equal to 0.1; (Santosa et al., 2018; Strangman et al., 2003). The GLM was fit to the long‐channel data—isolated by rejecting channels <20 mm or >40 mm. The design matrix for the GLM was generated by convolving a 3‐s boxcar function at each event‐onset time with the canonical haemodynamic response function (Glover, 1999). The boxcar function is shorter than the stimulus duration, informed by Luke, Larson, et al.'s (2021) demonstration that a 3‐s boxcar function maximised true positive rate for haemodynamic responses evoked by 5‐s auditory stimuli. The GLM also included all principal components of short‐detector channels to account for extracerebral and physiological signal components. Further, drift orders accounting for signal components up to 0.01 Hz were included as regression factors (Huppert, 2016). The GLM was performed with a lag‐1 autoregressive noise model, to account for the correlated nature of the fNIRS signal components. Individual beta estimates were then averaged for each brain region of interest (ROI), weighted by the standard error.

##### Second level

To predict the amplitude of measured haemodynamic responses in response per condition per ROI at the group‐level, linear mixed‐effects models were fit to estimates extracted from the first‐level analysis (*N* = 660 for each HbO and HbR), using the lme4 R package. Models for each HbO and HbR were constructed using a forward stepwise approach (Bates, Kliegl, et al., 2015), that is, sequentially adding predictor terms and testing whether each added term accounted for a significantly greater proportion of the variance using a likelihood ratio test. Model construction began with random intercepts for *Participants* to account inter‐participant variability. Next, random coefficients (slopes of a categorical variable) of *Condition* per *Participant* were added to account for individual differences between the five levels of *Condition* within each participant. Finally, each *ROI*, *Condition*, and the interaction between *ROI* and *Condition* were added as fixed effects. For our second fNIRS session where behavioural accuracy was re‐assessed, fixed effects of *Accuracy* and the interaction between *ROI*, *Condition*, and *Accuracy* were also assessed. The intercept was suppressed to compare each predictor term against zero, that is, to determine whether the predicted amplitude of a haemodynamic response is significantly different from zero for a given ROI and condition (Santosa et al., 2018). Models and estimates for each ROI‐condition pair are presented in Supplementary Materials S3 and S4. Influential data points were identified using Cook's distance (Cook, 2011) and subsequently excluded. Unstandardised beta coefficients are reported, as the outcome variables are in the same units and thus, no standardisation is needed for comparison of predictors across models. To describe the goodness of fit of each model, marginal and conditional *R* ^2^ values are reported together as *R* ^2^_*m/c*_ for each model using the MuMIn R Package (version 1.43.17; Bartoń, 2020). Hypothesis testing was conducted using the emmeans R package (version 1.4.2; Lenth, 2021) to contrast relevant group‐level estimates of haemodynamic response amplitude. Contrasts were corrected for multiple comparisons using the false discovery rate procedure (Benjamini & Hochberg, 1995). All analysis code is available on https://osf.io/352qc/.

## 3 RESULTS

### 3.1 Attenuating variations in F0 reduces accuracy with which vocal emotions are recognised, albeit with emotion‐specific effects

Initially, we investigated the extent to which listeners weight acoustic features that provide cues to vocal emotions by attenuating variations in F0, intensity, and speech rate, either individually or in pairs. Previous investigations of cue‐weighting in vocal emotions either attenuated variations in individual acoustic features to observe how reducing the information provided by cues impairs accuracy (Gilbers et al., 2015; Luo et al., 2007) or attenuated variations in pairs of acoustic features to quantify listeners' abilities to make use of individual acoustic features providing potentially informative cues. Specifically, they provide preliminary evidence that when F0 information is missing, listeners' may be able to use intensity and/or speech rate cues to glean emotional meaning (e.g., Everhardt et al., 2020; Hegarty & Faulkner, 2013; Metcalfe, 2017), and that intensity cues may be more reliable than speech‐rate cues in doing so (e.g., Marx et al., 2015; Peng et al., 2012). Based on these reports, we hypothesise that the accuracy with which vocal emotions are identified is reduced as potential cues, ordered from least to most impactful, are rendered uninformative, that is, intensity and speech‐rate cues combined, then F0 cues alone, followed by F0 and intensity cues combined and, finally, F0 and speech‐rate cues combined. To test this hypothesis, we compared listeners' accuracy for each speech condition against the previous condition using sliding difference contrasts in GLMMs fit first for all the speech conditions together and then for each individual emotion (Supplementary Table S2a).

**FIGURE 2:**
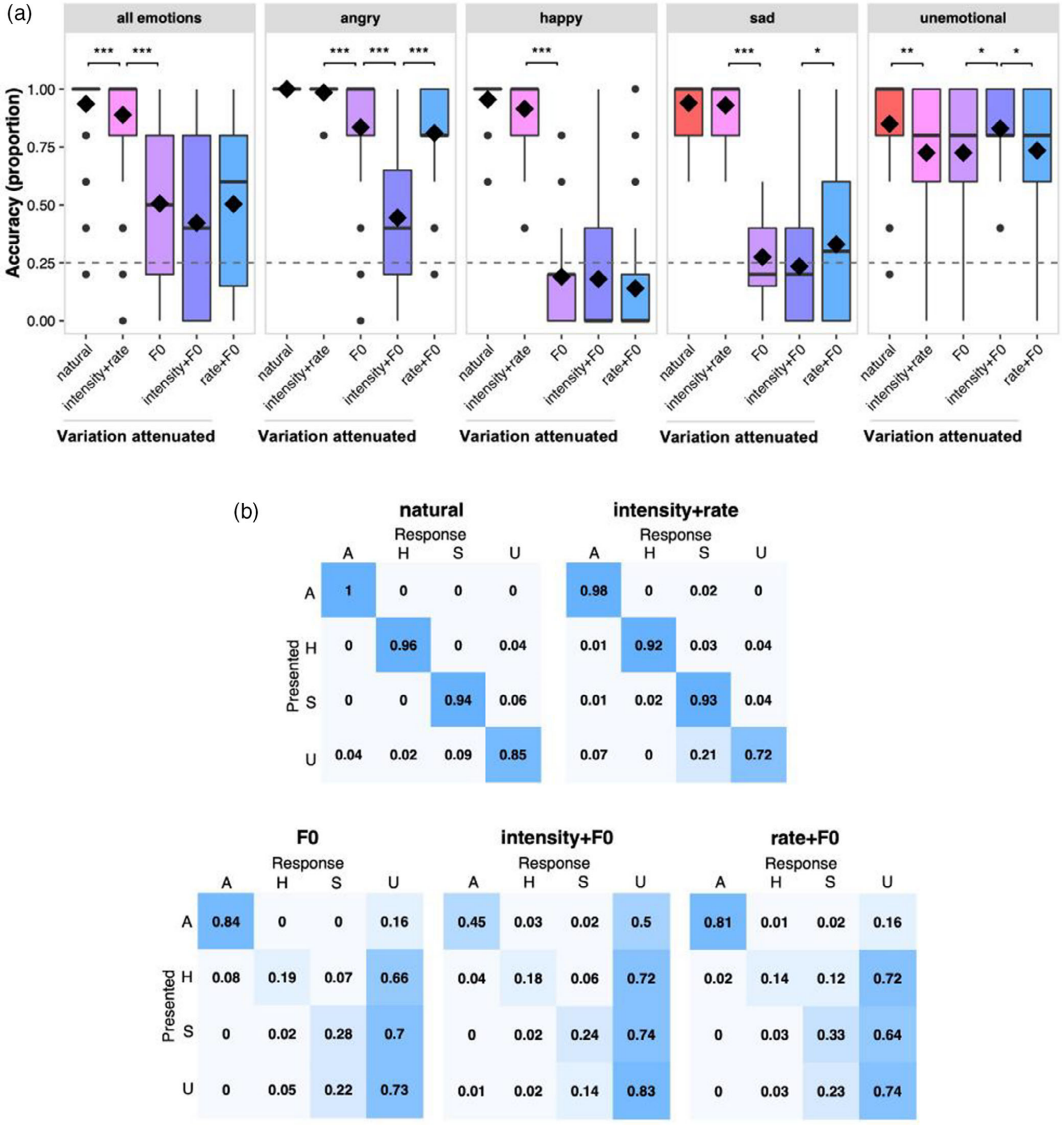
(a) Accuracy, overall and for each emotion per speech condition. Solid bar indicates median, diamond indicates mean, the box includes inter‐quartile range (IQR), whiskers indicate values within 1.5 times the IQR above the 75th or below the 25th percentile, dots indicate values outside 1.5 times the IQR. Significance (***p < .001, **p < .01, *p < .05) in each panel reflects outputs for each respective model, detailed in Supplementary Table S2a,b) The distribution of confusions—incorrect responses—across the response alternatives, as proportions. Top‐left to bottom‐right diagonal shows correct responses. A = *angry*, H = *happy*, S = *sad*, U = *unemotional*. Darker blue indicates greater proportion of responses.

We first compared the overall accuracy—for *happy*, *sad*, *angry*, or *unemotional* speech—with which listeners recognise emotions in speech in natural and manipulated speech (Figure 2a, left‐most panel). Accuracy with which emotions were recognized was reduced when variations in intensity and rate were simultaneously attenuated relative to the *natural* condition in which variations in F0, intensity, and speech rate were intact (*natural* vs. *rate* + *intensity*, *β* = .72, *p* < .001). Attenuating variations in F0 provided for reduced accuracy relative to simultaneously attenuated variations in intensity and rate (*rate* + *intensity* vs. *F0*, *β* = 2.84, *p* = .001). The mean reduction in proportion of correct responses for *natural* versus *rate* + *intensity* (0.05) relative to *rate* + *intensity* versus *F0* (0.38) suggests listeners were able to exploit intensity and speech rate cues to identify vocal emotions accurately, but that they relied more heavily on F0 cues. Attenuating variations in intensity and F0 simultaneously did not reduce accuracy any more than did attenuating F0 variations alone (*F0* vs. *intensity* + *F0*, *β* = .47, *p* = .381), and attenuating variations in speech rate and F0 concurrently did not reduce accuracy any more than did attenuating variations in intensity and F0 together (*intensity* + *F0* vs. *rate* + *F0*, *β* = .45, *p* = .424). This confirms that listeners were not able to make use of intensity cues when F0 cues were uninformative or speech rate cues when both intensity and F0 cues were uninformative (Juslin & Laukka, 2001; Scherer et al., 2003). The combination of intact intensity and speech‐rate cues supported accurate recognition of vocal emotion, but only when F0 cues were also intact. Together, our data suggest listeners rely most heavily on F0 cues and are limited in their ability to make use of intensity and speech‐rate cues to compensate for uninformative F0 cues.

We next assessed whether this was true for each investigated emotion individually by fitting a GLMM for each emotion separately. For *angry* speech, attenuating variations in intensity and speech rate did not reduce accuracy significantly (*natural* vs. *rate* + *intensity*, *β* = 15.69, *p* = .721), meaning that intact F0 cues alone were sufficient to support accurate recognition of *angry* speech. Accuracy fell when F0 variations were attenuated relative to when variations in intensity and speech rate were simultaneously attenuated (*rate* + *intensity* vs. *F0*, *β* = 3.10, *p* < .001), indicating listeners rely more heavily on F0 cues alone than on intensity and speech‐rate cues combined to recognise *angry* speech. Accuracy was further reduced when intensity and F0 variations were concurrently attenuated relative to when only variations in F0 were attenuated (*F0* vs. *intensity* + *F0*, *β* = 2.46, *p* < .001), indicating listeners can make use of intensity cues to recognise *angry* speech if F0 cues are uninformative. Further, listeners were more accurate in judging speech to be *angry* with attenuated variations in speech rate and F0 combined relative to attenuated variations in intensity and F0 combined (*intensity* + *F0* vs. *rate* + *F0*, *β* = −2.24 *p* < .001), suggesting they were not able to make use of speech‐rate cues when F0 and intensity cues were uninformative. Nevertheless, while listeners afforded most weight to F0 cues in judging speech to be *angry*, mean performance was above chance for all manipulations of *angry* speech; listeners were able to make use of intensity cues, but not speech‐rate cues, to recognise *angry* successfully when F0 cues were attenuated. The most common incorrect response to *angry* speech with attenuated F0 variations was *unemotional* (see confusion matrices in Figure 2b). The high accuracy for angry across all conditions is likely attributable to the overall intensity cues which, for this specific assessment, were not normalised across emotions. However, subsequent behavioural assessments, and fNIRS recordings, were made with intensity cues normalised. Voice‐quality cues such as soft or strained sounding voice (Laukkanen et al., 1997; Leitman et al., 2010; Patel et al., 2011; Waaramaa et al., 2010; Yanushevskaya et al., 2013), or dissonance cues associated with timbre may also have contributed to the ability of listeners to identify *angry* emotions in speech with attenuated F0 cues.

For *happy* speech, F0 cues alone were sufficient to support recognition of *happy* speech, with listeners able to identify *happy* unaffected by the simultaneous attenuation of variations in intensity and speech rate (*natural* vs. *rate* + *intensity*, *β* = .85, *p* = .680). Performance, however, was reduced when variations in F0 alone were attenuated relative to variations in intensity and speech rate combined (*rate* + *intensity* vs. *F0*, *β* = 6.08, *p* < .001). Further, listeners did not make use of intensity cues when F0 cues were uninformative (*F0* vs. *intensity* + *F0*, *β* = .1, *p* = .752) or speech‐rate cues when both intensity and F0 cues were uninformative (*intensity* + *F0* vs. *rate* + *F0*, *β* = .44, *p* = .188). Recognition of *happy* was at chance level for conditions in which variations in F0 were attenuated, where listeners most commonly misclassified *happy* for *unemotional*. Happy speech was also incorrectly judged to be *angry* or *sad* were, albeit with lower probability than *unemotional*, when F0 and rate variations were simultaneously attenuated (Figure 2b).

The ability to recognise *sad* speech was not altered by the combined attenuation of variations in intensity and speech rate (*natural* vs. *rate* + *intensity*, *β* = .19, *p* = .666), indicating that intact F0 cues alone support the successful recognition of *sad* speech. Relative to attenuated variations in intensity and speech rate simultaneously, attenuating variations in F0 reduced the accuracy with which *sad* speech was correctly identified (*rate* + *intensity* vs. *F0*, *β* = 4.73, *p* < .001). Relative to attenuated variations in F0 alone, accuracy with which *sad* speech was correctly identified was not reduced by simultaneous attenuation of variations in intensity and F0 (*F0* vs. *intensity* + *F0*, *β* = .27, *p* = .299); that is, listeners did not make use of intensity cues when F0 cues were attenuated. Accuracy was greater, however, when variations in speech rate and F0 combined were attenuated relative to when variations in intensity and F0 combined were attenuated (*intensity* + *F0* vs. *rate* + *F0*, *β* = −.61, *p* = .017). For *sad* speech, accuracy was reduced to near chance level in all speech conditions with uninformative F0 cues—most commonly mistaken for *unemotional* (Figure 2b)—indicating that listeners relied mainly on these cues to recognise *sad* speech (Metcalfe, 2017; Pell, 1998), but that they may make use of intensity cues when F0 and speech‐rate cues are simultaneously uninformative.

Finally, for *unemotional* speech, the model's fixed‐effect structure explained only 4% of the variance in the data (see *R* ^2^_*m*_ in Supplementary Table S2a) suggesting a poor model fit and meaning that significant effects should be interpreted with great caution. Relative to natural speech, simultaneously attenuating variations in intensity and speech rate reduced listeners' abilities to recognise *unemotional* speech (*natural* vs. *rate* + *intensity*, *β* = 1.84, *p* < .001). Uninformative F0 cues did not impact listeners' abilities to identify *unemotional* speech; accuracy was similar when variations in intensity and speech‐rate were simultaneously attenuated and when variations in F0 were attenuated (*rate* + *intensity* vs. *F0*, *β* = −.01, *p* = .990). However, accuracy increased when variations in intensity and F0 combined were attenuated relative to variations in F0 alone (*F0* vs. *intensity* + *F0*, *β* = −.85, *p* = .003), and was reduced when variations in F0 and speech rate combined were attenuated relative to attenuations of F0 and intensity variations combined (*intensity* + *F0* vs. *rate* + *F0*, *β* = .78, *p* = .006). For *unemotional* speech, accuracy was well above chance for all manipulations, with the most common misidentification for all being *sad* (Figure 2b).

Our data demonstrate that rendering F0 cues uninformative by attenuating variations in F0 reduces the accuracy with which listeners proficient at recognising emotions in natural speech can identify vocal emotions. To a lesser degree, rendering intensity and speech‐rate cues simultaneously uninformative also reduces the accuracy with which listeners recognise vocal emotions. The larger reduction in accuracy that arises when F0 cues are uninformative supports the view that listeners weight F0 most heavily in their decisions, consistent with existing evidence of the central role of F0 cues in recognising vocal emotions (Juslin & Laukka, 2001; Metcalfe, 2017; Scherer et al., 2003).

### 3.2 fNIRS‐mediated cortical haemodynamic responses fail to distinguish between different vocal emotions

We next examined the cortical activity evoked by individual emotions (*angry*, *happy*, *sad*, and *unemotional*) conveyed in natural speech using fNIRS, with the aim of determining whether ROI‐based analysis capture signatures of individual emotions, or whether only emotional relative to unemotional speech is captured in the response. Functional neuroimaging studies consistently implicate the right superior temporal cortex (STC) in the processing of vocal emotions, but the reported cortical haemodynamic activity evoked by individual emotions, such as *angry*, *happy*, or *sad*, varies considerably between studies. *Angry* speech has been most thoroughly investigated with fMRI and compared to *unemotional* or neutral speech is reported to activate the right or bilateral STC (Frühholz & Grandjean, 2012; Grandjean et al., 2005; Quadflieg et al., 2008), left, right, or bilateral IFG (Gruber et al., 2020; Korb et al., 2014; Quadflieg et al., 2008; Vytal & Hamann, 2010), and left or bilateral MFG (Bach et al., 2008; Kotz et al., 2013; Sonkaya & Bayazıt, 2018; Zhen et al., 2021). Investigations of *happy* relative to *unemotional* speech report increased haemodynamic activity in either left IFG (Frühholz, van der Zwaag, et al., 2016; Kotz et al., 2013; Zhang et al., 2018) or right STC (Buchanan et al., 2000; Leitman et al., 2010; Vytal & Hamann, 2010), while *sad* speech elicits increased haemodynamic activity in either right or left MFG relative to *unemotional* (Anuardi & Yamazaki, 2019; Buchanan et al., 2000; Kotz et al., 2013; Vytal & Hamann, 2010). The lack of consistency between studies provides substantial evidence that functional imaging techniques such as fMRI and fNIRS are unlikely to be highly sensitive to cortical representations of individual emotions.

**FIGURE 3:**
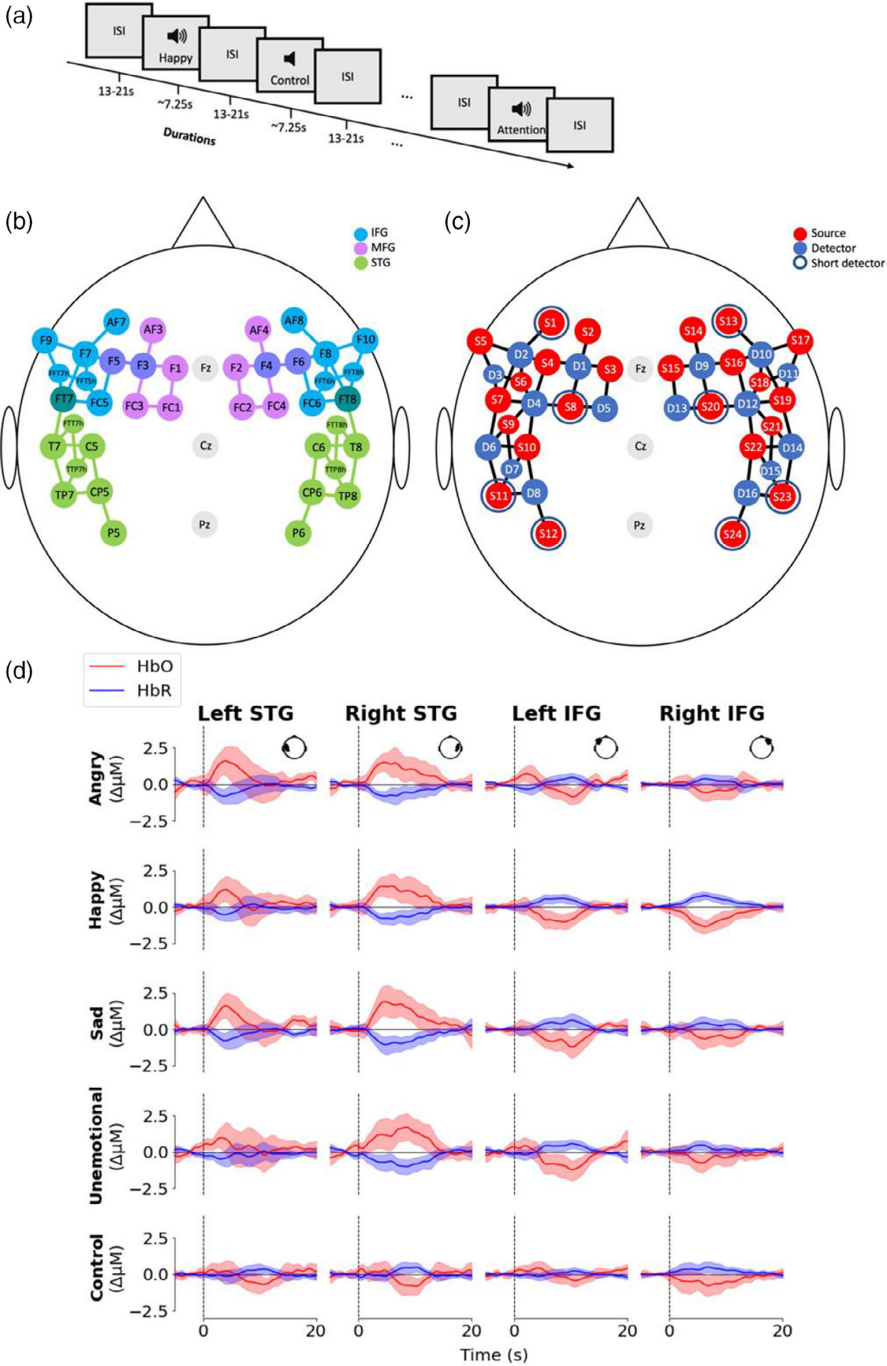
(a) Schema of functional near‐infrared spectroscopy (fNIRS) listening task. ISI = inter‐stimulus‐interval, loud‐speaker symbol = listening trial. (b) Positions of optodes in 10/10 and 5/10 systems with colour‐coded regions of interest (ROIs); STG = superior temporal gyrus, IFG = inferior frontal gyrus, MFG = middle frontal gyrus. IFG and MFG overlap at F3, F5, F4, and F6. IFG and STG overlap at FT7 and FT8. (c) Arrangement of sources, detectors, and short‐channel detectors. (d) Grand average waveforms for condition and ROI. Solid line = mean, shaded area = 95% confidence interval. The position of the optodes in each ROI represented in the inset head shape (viewed from above). Generation of waveforms detailed in Supplementary Material S3, distribution of SCIs presented in Supplementary Figure S3a, and MFG waveforms in Supplementary Figure S3b.

Here, we employ an ROI approach to assess the involvement of bilateral STG, IFG, and MFG reporting both chromophores (HbO and HbR) and using more densely placed channels, relative to previous fNIRS investigations, thereby increasing coverage of the cortex (Figure 3). Further, we employ the necessary regression of extracerebral and other signal components using short channels, often lacking in earlier investigations, to search for cortical signatures of individual emotions. If fNIRS is sensitive to cortical haemodynamic signatures of individual emotions, comparisons with *unemotional* will yield increased haemodynamic activity in right STG for *angry*, right STG and left IFG for *happy*, and bilateral MFG for *sad*. More generally, bilateral STG will exhibit increased haemodynamic activity evoked by speech, as compared to silence, and vocal emotions will evoke greater haemodynamic activity in right, relative to left, STG.

**FIGURE 4:**
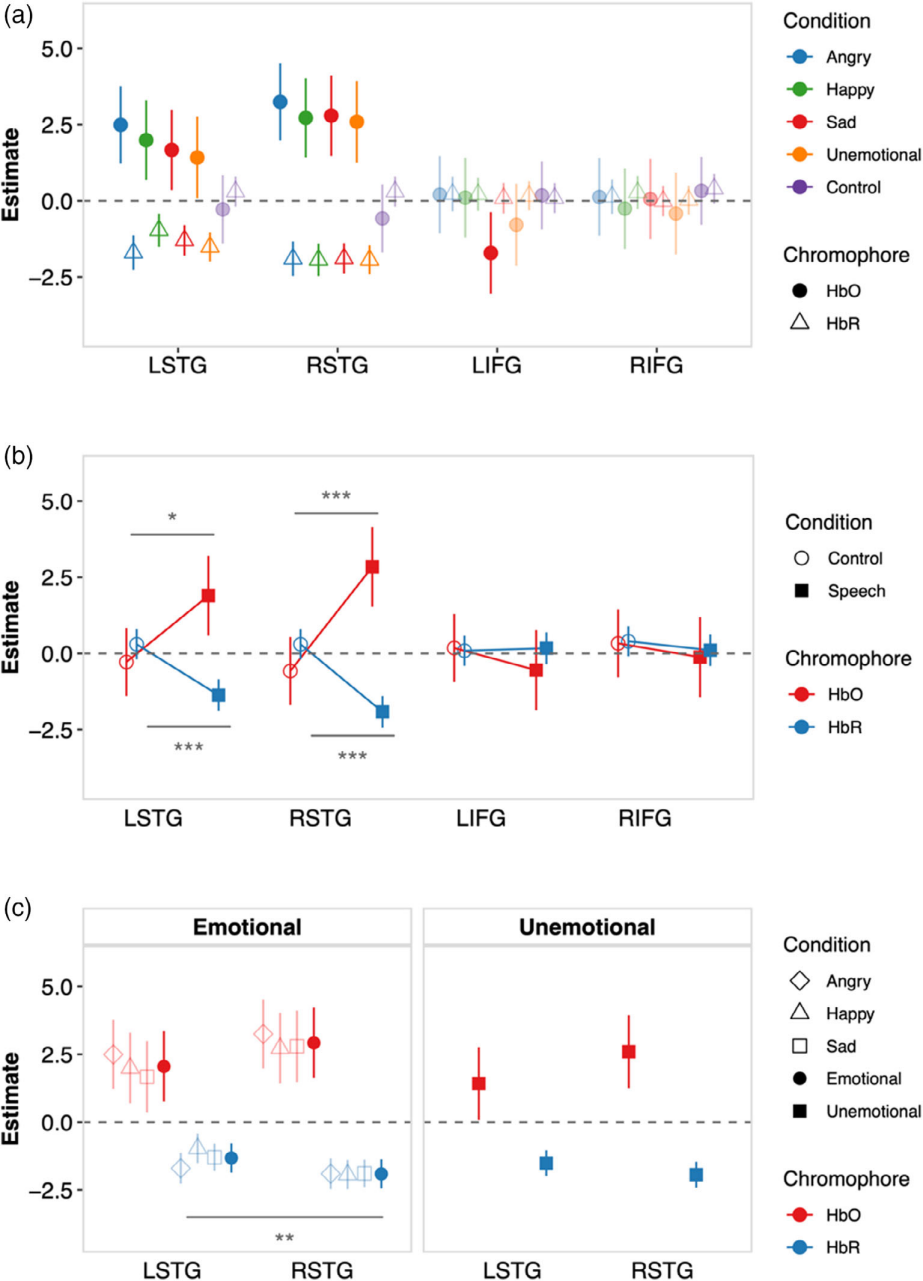
(a) Group‐level response estimates for each region of interest (ROI), condition and chromophore, extracted from Models 6 and 7. Darker symbols = significantly different from zero (*p* < .05), transparent symbols = non‐significant difference from zero. (b) Contrasts between speech and *control* conditions for each ROI. (c) Contrasts between left and right superior temporal gyrus (STG) for each emotional and *unemotional* speech; individual emotions included as transparent symbols for reference. Error bars indicate 95% confidence intervals, averaged across aggregated conditions where appropriate in (b) and (c). Significance for contrasts in (b) and (c), *p < .05, **p < .01, ***p < .001.

To ascertain the presence of auditory responses to speech, we contrasted estimates of the amplitudes of haemodynamic responses extracted from the second‐level, quantitative GLM analysis between speech (*angry*, *happy*, *sad*, *unemotional* aggregated) and the *control* (silent) conditions, demonstrating that speech stimuli evoke higher concentrations of HbO in left (*p* = .030) and right (*p* < .001) STG, relative to silence (Figure 4b, Table 2). Congruently, concentrations of HbR were significantly lower for speech compared to silence in STG bilaterally (*p* < .001). To determine whether cortical haemodynamic responses assessed using fNIRS are influenced by individual emotions, and therefore might yield individual cortical signatures for each emotion, we contrasted the estimates of the amplitude of haemodynamic response for each *angry* versus *unemotional*, *happy* versus *unemotional*, and *sad* versus *unemotional*, speech. No significant differences were observed in any ROI for either HbO or HbR for *angry*, *happy*, or *sad* relative to *unemotional* (all *p* > .05; Table 2), indicating that fNIRS cannot capture cortical signatures of individual emotions using an ROI approach. A subsequent channel‐wise analysis corroborates these findings (see Figure S3c, Table S3c,d—Supplementary Material). Notably, bilateral MFG showed no significant change in haemodynamic activity to any emotion in the GLM analysis, although a strong physiological response was evident in the generated waveforms (see Supplementary Figure S3b).

**TABLE 2:** Experiment 2: Estimates from planned contrasts between group‐level estimates of haemodynamic response amplitude.

Contrast		HbO					HbR				
		*β*	*SE*	*CI*	*t*	*p*	*β*	*SE*	*CI*	*t*	*p*
Speech vs. control	LSTG	**2.17**	**0.70**	**−0.04, 4.39**	**3.13**	**.030**	**−1.67**	**0.29**	**−2.60, −0.75**	**−5.78**	**<.001**
	RSTG	**3.42**	**0.70**	**1.20, 5.63**	**4.92**	**<.001**	**−2.22**	**0.29**	**−3.14, −1.30**	**−7.68**	**<.001**
	LIFG	−.73	0.70	−2.94, 1.49	−1.05	.848	.08	0.29	−0.84, 1.00	0.28	.995
	RIFG	−.45	0.70	−2.67, 1.77	−0.65	.848	−.29	0.29	−1.21, 0.63	−1.01	.995
	LMFG	−.41	0.70	−2.63, 1.80	−0.59	.848	−.01	0.29	−0.93, 0.91	−0.03	.995
	RMFG	−.25	0.70	−2.46, 1.97	−0.36	.928	−.57	0.29	−1.49, 0.35	−1.97	.338
Angry vs. unemotional	LSTG	1.07	0.91	−1.84, 3.98	1.18	.848	−.19	0.34	−1.27, 0.89	−0.56	.995
	RSTG	.65	0.91	−2.26, 3.56	0.72	.848	−.04	0.34	−1.04, 1.11	0.11	.995
	LIFG	.99	0.91	−1.92, 3.90	1.09	.848	.06	0.34	−1.02, 1.14	0.17	.995
	RIFG	.54	0.91	−2.37, 3.45	0.60	.848	.11	0.34	−0.97, 1.19	0.33	.995
	LMFG	.59	0.91	−2.32, 3.50	0.65	.848	−.14	0.34	−1.22, 0.93	−0.42	.995
	RMFG	.89	0.91	−2.01, 3.80	0.98	.848	−.22	0.34	−1.30, 0.85	−0.65	.995
Happy vs. unemotional	LSTG	.57	0.92	−2.39, 3.53	0.62	.848	.54	0.35	−0.58, 1.67	1.53	.658
	RSTG	.13	0.92	−2.83, 3.08	0.14	.928	.00	0.35	−1.11, 1.11	0.01	.995
	LIFG	.89	0.92	−2.07, 3.85	0.17	.848	.05	0.35	−1.07, 1.17	0.15	.995
	RIFG	.16	0.93	−2.82, 3.14	0.17	.928	.25	0.35	−0.86, 1.36	0.71	.995
	LMFG	.28	0.92	−2.68, 3.24	0.30	.928	−.02	0.35	−1.13, 1.09	−0.05	.995
	RMFG	.13	0.92	−2.82, 3.09	0.14	.928	−.11	0.35	−1.22, 1.00	−0.31	.995
Sad vs. unemotional	LSTG	.25	0.75	−2.09, 2.59	0.34	.928	.21	0.32	−0.81, 1.23	0.66	.995
	RSTG	.20	0.75	−2.14, 2.54	0.27	.928	.05	0.32	−0.97, 1.07	0.15	.995
	LIFG	−.92	0.76	−3.30, 1.45	−1.22	.848	−.08	0.32	−1.10, 0.94	−0.24	.995
	RIFG	.47	0.75	−1.87, 2.82	0.63	.848	−.03	0.32	−1.05, 1.00	−0.10	.995
	LMFG	.37	0.75	−1.98, 2.71	0.49	.902	−.13	0.32	−1.15, 0.89	−0.40	.995
	RMFG	.03	0.75	−2.32, 2.37	0.04	.972	−.33	0.32	−1.35, 0.69	−1.01	.995
RSTG vs. LSTG	Emotional	.87	0.42	−0.44, 2.18	2.08	.334	**−.59**	**0.18**	**−1.14, −0.04**	**−3.33**	**.008**
	Unemotional	1.18	0.73	−1.09, 3.44	1.62	.688	−.43	0.30	−1.37, 0.52	−1.41	.700

We next contrasted estimates of response amplitude in left and right STG for emotional (*angry*, *happy*, *sad* together) and for *unemotional* speech. For HbO, response estimates for emotional and *unemotional* speech conditions were numerically higher in right, compared to left, STG, but these differences were not statistically significant (both *p* > .05; Figure 4c, Table 2). For HbR, a right‐lateralisation of haemodynamic activity was observed for emotional speech with reduced HbR in right, compared to left, STG (*p* = .008), but not for *unemotional* speech (*p* = .700), supporting the hypothesis that emotional speech evokes haemodynamic activity predominantly in right, compared to left, STG.

Our data suggest that haemodynamic responses measured using fNIRS are sensitive to speech‐evoked activation of the STG bilaterally, and the right‐lateralisation of STG for emotional speech. However, the similarity of the amplitude of the speech‐evoked responses for all emotions shows that fNIRS, even measured with the relatively high channel density and short‐channel regression we employed here, does not capture any differences in emotional meaning within cortical representations of speech. This suggests that fNIRS is better suited to investigating components of vocal emotion related to speech per se, such as the contributions of acoustic cues to speech processing, rather than to assessing the identity of specific emotions (i.e., *happy*, *sad*) conveyed in that speech.

### 3.3 Behavioural accuracy in recognising vocal emotions predicts haemodynamic responses during passive listening

**FIGURE 5:**
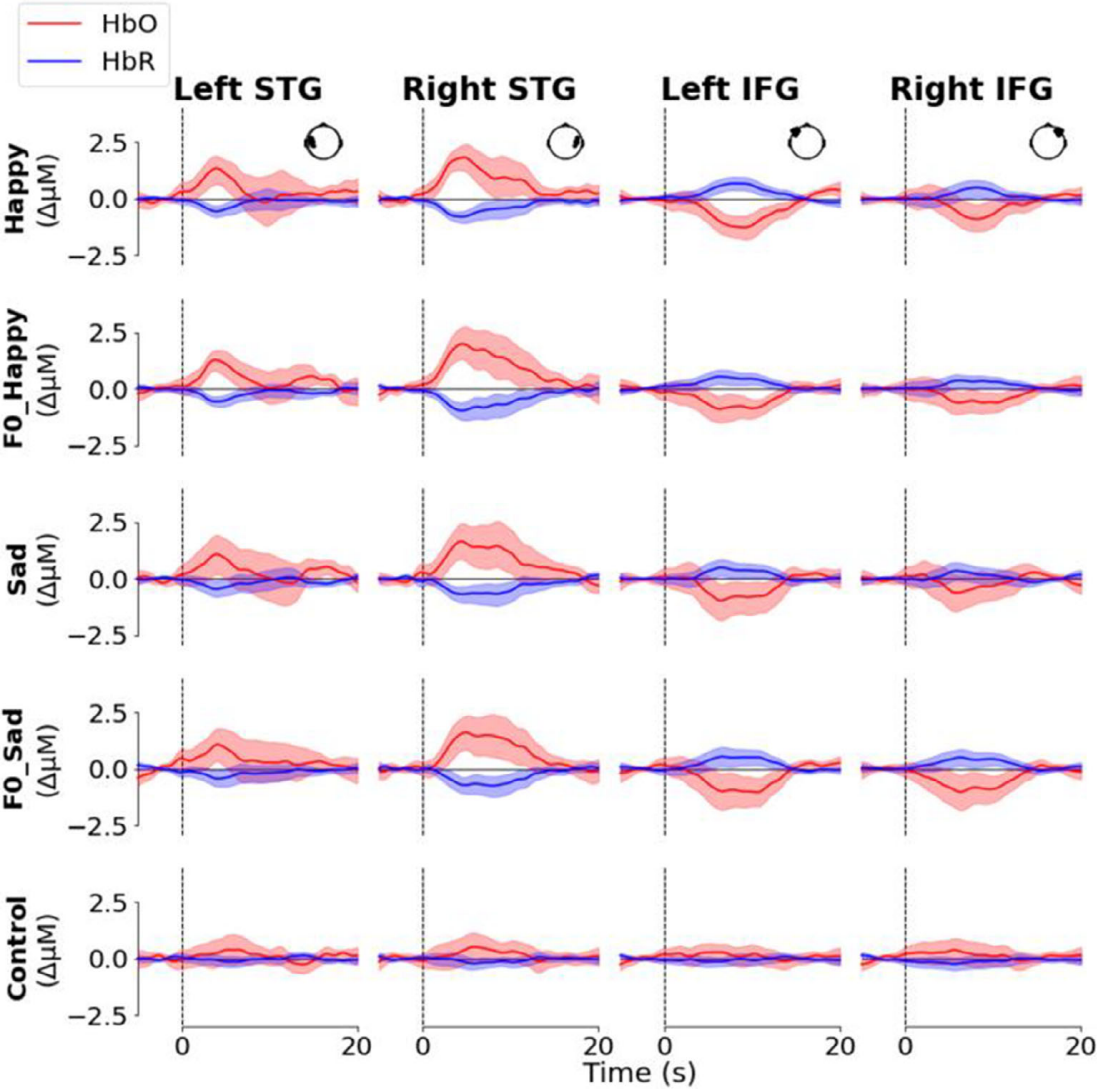
Grand average waveforms for all conditions (rows) and regions of interest (ROIs) (columns). Solid line = mean, shading = 95% confidence interval. Inset head shape in the top right corner of each ROI panel (viewed from above) shows the position of the optodes on the head. Generation of waveforms detailed in Supplementary Material S3, SCIs in Supplementary Figure S4a, and MFG waveforms in Supplementary S4b.

Having established that listeners' abilities to recognise emotions in speech is strongly reduced when F0 cues are rendered uninformative, and that differences in cortical activation between emotions are unlikely to be detected using fNIRS, we next investigated whether activity evoked in right and left STG (and IFG) by vocal emotions is influenced by the availability or lack of rich F0 cues (Figure 5). Our rationale for doing so is that any observed cortical activity may constitute a potential biomarker for behavioural deficits in recognising emotions conveyed in speech, assessed here in normal‐hearing listeners challenged with speech containing uninformative F0 cues.

**FIGURE 6:**
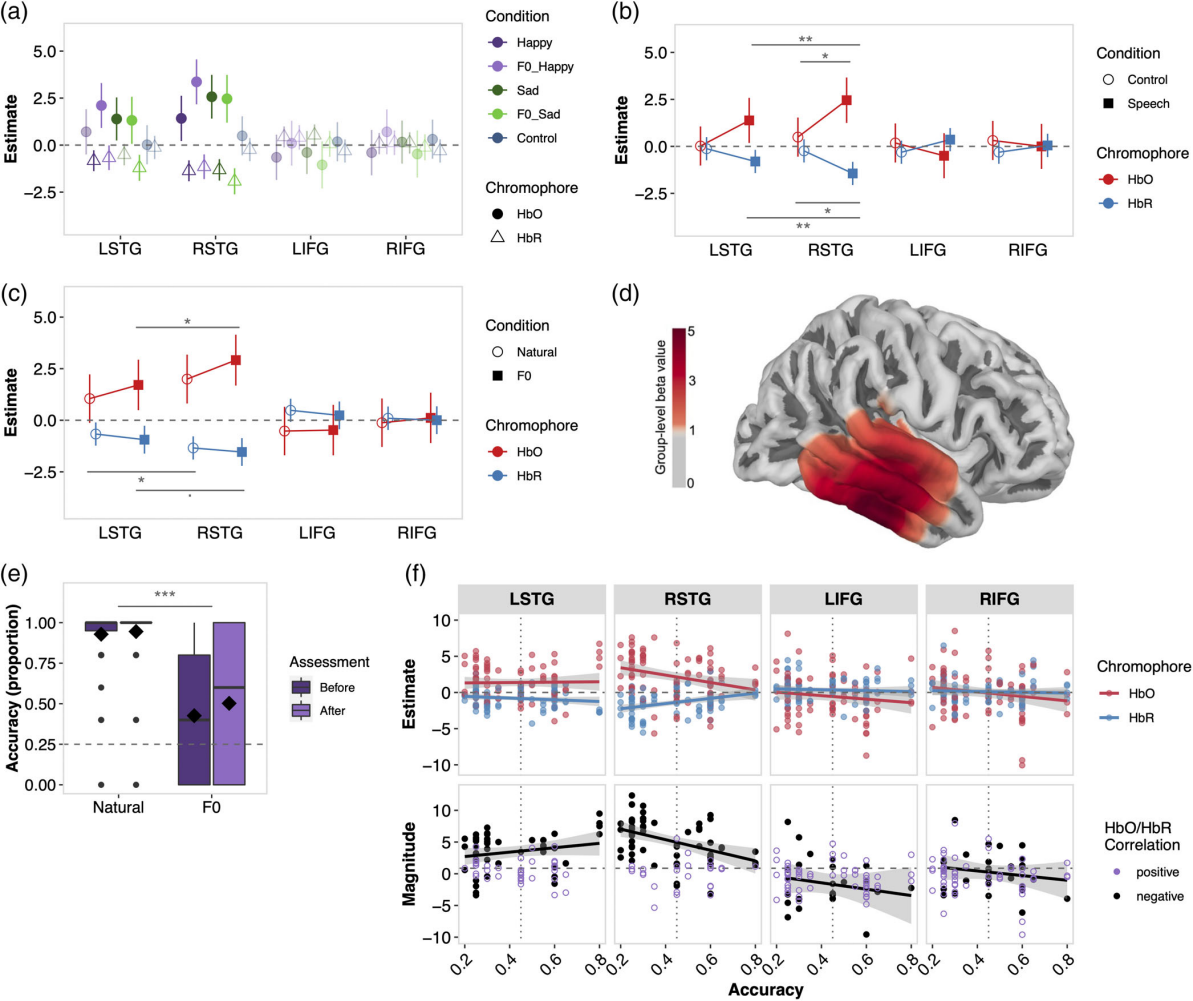
(a) Group‐level response estimates for each region of interest (ROI), speech condition and chromophore. Darker and transparent symbols indicate significant and non‐significant differences from zero, respectively. (b) Contrasts *control* versus aggregated speech conditions for each ROI, as well as left versus right superior temporal gyrus (STG) for aggregated speech conditions. (c) Contrasts vocal emotions conveyed in natural speech (*happy* + *sad*) vs. speech with uninformative F0 cues (*F0_happy* + *F0_sad*), within each ROI, as well as between left versus right STG. Error bars indicate 95% confidence interval, per response estimate in (a), and averaged across aggregated conditions in (b) and (c). d) Projection of right‐hemisphere group‐level estimates (HbO) for all speech conditions onto cortex. (e) The proportion of correct responses aggregated across emotion per condition and test time (before or after functional near‐infrared spectroscopy [fNIRS] session 2). Diamond indicates mean. (f) Relationship between accuracy of emotion recognition before the second fNIRS recording with attenuated F0 variations and haemodynamic activity per ROI, per chromophore (upper) and as a measure of the magnitude of the difference between Hb and HbR (lower); RSTG relationship significant (*p* < .05 for HbO, HbR, and magnitude of differences based on negatively correlated estimate pairs). Significance for contrasts in (b), (c), (e), *p < .05, **p < .01, ***p < .001.

We proceeded to re‐assess listeners' abilities to recognise *angry*, *happy*, *sad*, and *unemotional* speech with and without attenuated F0 cues in a new group of listeners (many of whom completed fNIRS session 1, but whose emotion recognition abilities were still unknown) before and after a set of fNIRS recordings were made (Figure 6e). As before, listeners were highly proficient at recognising emotions conveyed in natural speech, and significantly less so when variations in F0 were attenuated (*β* = −4.05, *p* < .001). No significant difference was observed between behavioural performance assessed before and after the fNIRS recording (*β* = .49, *p* = .178; Supplementary Table S4a), indicating that exposure to the subset (*happy* and *sad*) of emotions in speech with and without attenuated F0 cues during the fNIRS recording did not significantly alter the accuracy with which listeners recognised the set of four emotions presented in the subsequent behavioural assessment.

Contrasts confirmed that cortical activations to vocal emotions with natural and attenuated F0 cues (i.e., *happy*, *sad*, *F0_happy*, and *F0_sad* aggregated) were larger in STG bilaterally than in the silent *control* condition. Significant differences were observed for HbO (*p* = .021) and HbR (*p* = .019) in right STG, and left STG exhibited similar numerical trends although contrasts yielded non‐significant differences (both chromophores *p* > .05). No significant differences were observed between the *control* and speech conditions in left or right IFG for either chromophore (all *p* > .05; Table 3). Contrasting estimates of haemodynamic response amplitude in right STG with left STG (Figure 6b) the aggregated vocal emotions (*happy*, *sad*, *F0_happy*, and *F0_sad*) confirmed a right‐lateralisation of STG activity evoked by vocal emotions for both HbO (*p* = .009) and HbR (*p* = .003). This lateralisation held for natural speech (*happy* and *sad*), with significantly larger haemodynamic response amplitudes observed for right compared to left STG for HbR (*p* = .029), but not HbO (*p* = .095; Figure 6c and Table 3). For speech with attenuated variations in F0 (*F0_happy* and *F0_sad*), lateralisation was significant for HbO (*p* = .034) and approached significance for HbR (*p* = .056). So too, contrasting estimates of haemodynamic response amplitude in right STG with left STG (Figure 6b) the aggregated vocal emotions (*happy*, *sad*, *F0_happy*, and *F0_sad*) evoked enhanced haemodynamic activity in right STG relative to left by confirming a right‐lateralisation of STG activity evoked by vocal emotions for both HbO (*p* = .009) and HbR (*p* = .003). For natural speech (*happy* and *sad*), significantly larger haemodynamic response amplitudes were observed for right compared to left STG for HbR (*p* = .029), but not HbO (*p* = .095; Figure 6c and Table 3). For speech with attenuated variations in F0 (*F0_happy* and *F0_sad*) larger‐amplitude haemodynamic responses were observed for right compared to left STG for HbO (*p* = .034), but the difference only approached significance for HbR (*p* = .056). Overall, and consistent with the larger set of emotions included in fNIRS session 1, the data support the hypothesis that the haemodynamic activity evoked by vocal emotions in natural speech is larger in right than left STG (Beaucousin et al., 2007; Witteman et al., 2012), and that the same is true for vocal emotions in which F0 cues were rendered uninformative by attenuating variations in F0.

**TABLE 3:** Experiment 3: Estimates from planned contrasts between group‐level estimates of haemodynamic response amplitude.

Contrasts		HbO					HbR				
		*β*	*SE*	*CI*	*t*	*p*	*β*	*SE*	*CI*	*t*	*p*
Emotions vs. control	LSTG	1.36	0.61	−0.56, 3.28	2.22	.095	−.68	0.37	−1.84, 0.48	−1.85	.235
	RSTG	**1.96**	**0.62**	**0.04, 3.89**	**3.19**	**.021**	**−1.20**	**0.37**	**−2.37, −0.04**	**−3.26**	**.019**
	LIFG	−.68	0.61	0.04, 3.89	−1.11	.461	.67	0.37	−0.49, 1.83	1.82	.235
	RIFG	−.32	0.61	−2.60, 1.24	−0.52	.755	.36	0.37	−0.80, 1.53	0.98	.568
Natural vs. F0	LSTG	−.66	0.50	−2.20, 1.61	−1.34	.351	.27	0.27	−0.54, 1.09	1.03	.568
	RSTG	−.92	0.50	−2.47, 0.63	−1.83	.168	.20	0.27	−0.62, 1.01	0.74	.675
	LIFG	−.05	0.50	−1.58, 1.49	−0.09	.926	.24	0.27	−0.57, 1.06	0.92	.568
	RIFG	−.24	0.50	−1.77, 1.30	−0.48	.755	.09	0.27	−0.73, 0.92	0.35	.810
Happy_F0 vs. happy	LSTG	1.40	0.71	−0.79, 3.59	1.98	.137	.16	0.38	−1.00, 1.31	0.42	.803
	RSTG	**1.94**	**0.71**	**−0.24, 4.13**	**2.75**	**.034**	.22	0.38	−0.94, 1.37	0.58	.766
	LIFG	.75	0.71	−1.44, 2.94	1.06	.461	−.03	0.38	−1.18, 1.13	−0.07	.943
	RIFG	1.11	0.71	−1.08, 3.29	1.57	.255	.04	0.38	−1.13, 1.21	0.12	.943
Sad_F0 vs. sad	LSTG	−.07	0.77	−2.46, 2.32	−0.10	.926	−.70	0.46	−2.15, 0.74	−1.54	.353
	RSTG	−.11	0.78	−2.54, 2.32	−0.14	.926	−.61	0.46	−2.05, 0.83	−1.33	.449
	LIFG	−.66	0.77	−3.05, 1.73	−0.86	.555	−.46	0.46	−1.90, 0.98	−1.01	.568
	RIFG	−.64	0.77	−3.03, 1.76	−0.83	.555	−.23	0.46	−1.68, 1.21	−0.51	.776
RSTG vs. LSTG	Emotional	**1.08**	**0.30**	**0.15, 2.00**	**3.53**	**.009**	**−.64**	**0.17**	**−1.42, −0.13**	**−3.81**	**.003**
	Natural	.95	0.43	−0.36, 2.25	2.20	.095	**−.67**	**0.24**	**−1.39, 0.04**	**−2.86**	**.029**
	F0	**1.20**	**0.43**	**−0.10, 2.51**	**2.79**	**.034**	−.60	0.24	−1.31, 0.12	−2.53	.056

We next contrasted estimates of the amplitude of haemodynamic responses to vocal emotions conveyed in natural speech versus speech with attenuated F0 variations. These yielded no significant differences in either chromophore or any ROI between (both *p* > .05; Table 3; Figure 6c). However, further contrasts computed for *happy* vs. *F0_happy* and *sad* versus *F0_sad*, revealed higher concentrations of HbO in right STG for *F0_happy* relative to *happy* speech (*p* = .034) but not HbR (*p* = .766) or any other ROI for either chromophore (all *p* > .05). No significant differences were found for any ROI or chromophore for *sad* versus *F0_sad* speech (all *p* > .05; Table 3).

Although the enhanced activity in right STG for *happy* relative to *sad* speech with uninformative F0 cues was initially unexpected, closer examination of the acoustic profiles of the two emotions in the *natural* conditions indicates that *happy* speech showed more variance in F0 than *sad* speech, and the mean F0 for *happy* speech was further from the target value (217 Hz) at which the F0 was held constant than the mean for *sad* speech. We propose that the larger amplitudes in cortical haemodynamic responses in right STG observed for *happy* than *sad* speech when F0 cues are attenuated indicate a difference in prediction error (Friston, 2005; Stein et al., 2021), mediated by the relative distance between the original and manipulated acoustic profiles for each emotion. That is, we propose that the difference in richness of F0 cues between *happy* with and without attenuated F0 cues is larger than the difference between the two versions of *sad*, and that greater prediction error, observed in enhanced haemodynamic activity, likely reflects the relative reduction in the richness of F0 cues.

Finally, we also assessed the relationship between listeners' accuracy recognising emotions with attenuated variations in F0 cues and listeners' haemodynamic response amplitude in each ROI (Figure 6f, Models 12 and 13 slope estimates in Supplementary Table S4c). In right STG, the amplitude of the haemodynamic response decreased significantly with increasing *Accuracy* for both HbO (*p* = .030) and HbR (*p* = .003). For HbO, the amplitude of the haemodynamic response in left STG (*p* = .990) did not covary with *Accuracy* and decreased in IFG bilaterally, though not significantly (both *p* > .05), with increasing *Accuracy*. Bilaterally in IFG, HbR did not covary significantly with *Accuracy*, while HbR in left STG was not influenced by *Accuracy* (*p* = .331).

We also assessed whether this relationship held for the unsigned magnitude of the difference between HbO–HbR (mainly used in clinical studies to date, e.g., Kaynezhad et al., 2019; Kolyva et al., 2014). Here, larger difference values between negatively correlated HbO–HbR pairs are assumed to reflect true cortical activity and while positively correlated pairs do not. Our analyses revealed that the relationship between emotion recognition accuracy and HbO–HbR difference values takes the same pattern as observed for each HbO and HbR, regardless of whether model is fit to only negatively correlated difference values or all difference values: right STG activity increases with decreasing accuracy (Figure 6f, Supplementary Table S4d). Beyond the confirmation of our findings, we wish to highlight the value of the HbO–HbR difference as a conceptually straightforward metric for synthesising the HbO and HbR amplitudes into a single value. A compelling advantage of the HbO–HbR difference is the much‐needed oversight it provides into haemodynamic response patterns observed in individual participants, that is, the relative proportions of negatively and positively correlated pairs of estimates (see lower panel of Figure 6f).

## 4 DISCUSSION

Using fNIRS, we examined cortical responses of normal‐hearing listeners to vocal emotions in natural speech, to determine whether ROI‐based analysis of cortical processing of vocal emotions is influenced by the signatures of individual emotions. No differential cortical signature was observed between *angry*, *happy*, or *sad* speech, suggesting that even with the relatively high density of optical sources and detectors we employed, fNIRS is insensitive to differences in emotional prosody conveyed in speech. However, exploring the relationship between cortical haemodynamics and behavioural sensitivity to speech in which the dominant cue—variations over time in the fundamental frequency (F0)—for conveying emotion were attenuated, we found a significant relationship between cortical activation and the accuracy with which vocal emotions are recognized. Specifically, elevated fNIRS signals in right STG were associated with lower accuracy in recognised vocal emotions when F0 cues were attenuated. Together, the data suggest that increased neural resources might be recruited in situations where recognizing emotional cues conveyed in speech is challenging due to the reduction in variations in F0 over time that normally conveys prosodic features in speech. Listeners who are more able to recognize vocal emotions based on remaining cues (here, intensity and speech‐rate) showed overall lower haemodynamic responses.

### 4.1 Uninformative F0 cues reduce listeners' ability to recognise vocal emotions—All emotions sound unemotional

Our behavioural data confirm that listeners are proficient at recognising emotions in natural speech and that rendering F0 cues uninformative by attenuating variations in F0 reduces the accuracy with which listeners can identify vocal emotions and that, to a lesser degree, rendering intensity and speech‐rate cues simultaneously uninformative also reduces the accuracy with which listeners recognise vocal emotions. The large reduction in accuracy that arises when F0 cues are uninformative supports the view that listeners weight F0 most heavily in their decisions, consistent with existing evidence for the central role of F0 cues in recognising vocal emotions (Juslin & Laukka, 2001; Metcalfe, 2017; Scherer et al., 2003). The main consequence of rendering acoustic cues to vocal emotions uninformative is that listeners mistake all emotions (*angry*, *happy*, *sad*) for *unemotional* speech. This is consistent with previous reports (Luo et al., 2007; Metcalfe, 2017), as well as Metcalfe's (2017) suggestion that listeners select “neutral” or *unemotional* when cues are uninformative making emotion recognition more challenging. Metcalfe (2017) also suggests that listeners may use “neutral” or *unemotional* as a “not sure” response among the alternatives in a forced‐choice task, an interpretation at least consistent with the data presented here.

### 4.2 No evidence of cortical signatures of individual emotions using fNIRS

Importantly, we found that cortical activity generated by *angry*, *happy*, and *sad* speech did not differ significantly from that generated by *unemotional* speech in any ROI, indicating that fNIRS, using an ROI‐based approach, is not suited to the investigation of differences in the cortical representation of discrete emotions. To this end, fNIRS provides a means for investigating lower‐level components of emotion processing (acoustic cue perception and integration, as opposed to percept categorisation and recognition; Frühholz, Trost, & Kotz, 2016). Our data reveal a right hemisphere dominance for both natural speech and speech with attenuated variations in F0. In previous investigations of easily recognised vocal emotions, right‐lateralisation is attributed to a right‐hemisphere specialisation for decoding the melody of speech and changes in pitch (Flinker et al., 2019; Friederici & Gierhan, 2013; Poeppel, 2003). However, for vocal emotions to which F0 cues were uninformative, haemodynamic activity is unlikely to reflect the decoding of the F0 contour. Rather, increased haemodynamic activity observed in right relative to left STG may arise from the unnaturalness of a flattened F0 contour, by listeners attentively seeking to recognise the conveyed emotions, or a combination of these factors.

While our study is the first to investigate and report cortical haemodynamic responses to vocal emotions with uninformative F0 cues, the right‐dominant response to speech with uninformative F0 cues is consistent with evidence from one fMRI study of adult participants with non‐emotional speech (Meyer et al., 2004), evidence from EEG studies of right‐lateralisation of cortical activity to vocal emotions in CI listeners (Cartocci et al., 2021), and evidence that speech in which F0 cues are attenuated evokes increased neural activity in the right hemisphere (Liikkanen et al., 2007; Miettinen et al., 2011, 2012). Additionally, two fNIRS studies (Homae et al., 2007; Wartenburger et al., 2007) examining haemodynamic responses to child‐directed speech–characterised by increased variations in F0 and intensity (Fernald & Simon, 1984; Grieser & Kuhl, 1988)–report increased activity in right STG activity when variations in F0 are attenuated, consistent with the current findings. However, participants in those studies were 10‐month‐old infants (Homae et al., 2007) and 4‐year‐old children (Wartenburger et al., 2007), in whom morphology of cortical haemodynamic responses and sensitivity to vocal emotions are clearly not matured (Marcar et al., 2004; Morningstar et al., 2018; Nagels et al., 2020). The right‐lateralisation of STG activity may reflect the reduced usefulness of F0 cues in degraded speech, regardless of the emotional meaning assigned.

An alternative explanation for the right‐lateralisation of STG activity is that the sequence of tasks completed during the session (i.e., behavioural recognition assessed immediately before and after fNIRS recordings were made) could have led listeners to try to recognise the conveyed emotions explicitly during the listening task. The right‐lateralisation of STG activity is consistent with the enhancement of right‐hemisphere haemodynamic activity mediated by attention to vocal emotions (Frühholz et al., 2012; Kotz et al., 2013; Zhen et al., 2021), and accords with the suggestion (Kislova & Rusalova, 2009) that listeners who struggle to recognise emotions show cortical activation in EEG frequency bands related to increased attention and emotional tension (i.e., gamma and theta, respectively). It is plausible that both the alterations to the F0 cues and listeners' efforts to recognise emotions in the stimuli presented during fNIRS recordings contribute to the right‐lateralisation of activity to speech with attenuated F0 cues.

### 4.3 Ability to recognise emotions despite uninformative F0 cues correlates with right STG activity

An unexpected finding in our study is that the ability to recognise emotions in speech with attenuated variations in F0 is negatively correlated with the amplitude of haemodynamic responses in right STG; as accuracy with which listeners identify emotions in degraded speech decreased, haemodynamic activity in right STG increases (Figure 6f). This is at least consistent with Cartocci et al.'s (2021) report of a negative correlation between the right‐lateralisation of the EEG gamma frequency band and accuracy of emotion recognition, although a major difference is that all of the participants in that study, normal‐hearing listeners and users of CIs, heard natural speech and the relationship was not assessed separately for each group.

A plausible explanation for the increased haemodynamic activity in right STG when F0 cues are removed is that this reflects the perceptual sensitivity of individual listeners to attenuation of the F0 cues that convey emotions in speech; listeners who show reduced activity in right STG and higher behavioural accuracy may be able to make use of cues extracted from secondary acoustic features, such as intensity, speech rate, or voice quality, more successfully. It is also possible that listeners who are less sensitive to secondary acoustic features may expend more effort in this process. In our first behavioural assessment with a larger range of vocal emotions, listeners judged emotions conveyed in speech with systematically attenuated variations in F0, intensity, and/or speech rate, and considerable variability was observed in their abilities to recognise vocal emotions when F0 cues were uninformative. This variability, also evident when only *happy* and *sad* emotions were assessed, indicates that some listeners can overcome the challenge of uninformative F0 cues and exploit information conveyed by secondary cues to make correct judgements about vocal emotions. Although we did not test this directly, we posit that such listeners would show reduced right STG activity compared to listeners who cannot make use of other informative acoustic cues to recognise vocal emotions.

Individual differences in speech recognition scores for noise‐vocoded CI simulations, that is, speech with uninformative F0 cues, have been suggested to be mediated by the strategies that listeners adopt while learning to decode that speech. For example, taking a “top‐down” strategy, some listeners may seek to decode speech sounds from lexical and semantic information, while others employ a “bottom‐up” strategy, relying more on the acoustic‐phonetic cues (Başkent, Clarke, et al., 2016; Bhargava et al., 2014; McGettigan et al., 2014). NH listeners can recognise words in CI‐simulation pseudo‐sentences, indicating that the listeners can make use of the unattenuated acoustic‐phonetic information, that is, the “bottom‐up” strategy (Loebach et al., 2010). The stimuli in the assessments described here were pseudo‐words (to control for lexical and semantic information), which rhymed (to control for phonetic information), reducing the availability of information for top‐down processing. It is possible that listeners who made use of “bottom‐up” strategies in our study may have been more attuned to the unattenuated acoustic cues, enabling them to recognise vocal emotions more readily in speech with attenuated F0 cues. Listeners' individual abilities to extract emotional meaning from secondary cues, such as intensity and speech rate, are likely mediated by their abilities to perceive these cues, as well as their experience relying on the cues (Jasmin et al., 2019, 2021), and how well acclimatised they are to the altered speech signal (Davis et al., 2005).

Individual listeners' abilities to compensate for uninformative F0 cues by exploiting remaining informative acoustic cues may also mediate neuro‐metabolic demands in right STG. It is possible that these demands reflect inter‐listeners differences in prediction error—a measure of the distance between the predicted and true sensory input, as encoded by neurons (de Lange et al., 2018; Friston, 2005). Elevated cortical activity evoked by changes in the frequency of pure tones or in the frequency profile of sounds (Rubin et al., 2016; Stein et al., 2021) may reflect neural signature of a prediction error. We propose that listeners who rely more heavily on F0 cues (i.e., are less able to extract emotional meaning from other cues) exhibit evidence of increased prediction error in right STG when F0 cues are uninformative. The ability to exploit cues such as intensity, speech rate, and voice quality, likely promotes the updating of predictive models pertaining to the speech signal, facilitating accurate recognition and yielding reduced right STG activity. With this explanation in mind, we posit that the relationship between the behavioural and cortical responses to vocal emotions in speech without informative F0 cues shows promise as an objective measure of emotion recognition in speech with degraded F0 information.

### 4.4 Implications for processing of vocal emotions with hearing devices

Therapeutic hearing devices are used to mitigate some perceptual aspects of hearing loss. However, due to the limitations imposed by physiological changes caused by hearing loss or sometimes also limitations in technological aspects, the sounds transmitted are still different from that of acoustic normal hearing. HAs acoustically amplify sounds to compensate for reduced audibility, but there may still be distortions related to hearing loss beyond audibility, such as suprathreshold effects due to widened auditory filters or loudness recruitment (Başkent, 2006; Kortlang et al., 2016; Moore, 1996) which cannot be entirely compensated for by HAs. Further, HA signal processing needs to alter acoustic signals, for example, by applying dynamic range compression to match the reduced dynamic range of the HA user, and these features are applied with a time constant that may further affect the transmitted acoustic signal (Souza, 2002). The acoustic signals could further be affected due to practical aspects, such as limited bandwidth of amplification or microphone placement that is outside the pinna. CIs are for individuals who have little to no hearing ability of acoustic signals. As a result, CIs convert acoustic signals to trains of electrical pulses that directly stimulate the auditory nerve. Electric hearing mechanisms greatly differ to that of acoustic hearing, and limitations inherent to electric stimulation result in a heavily compressed intensity range and limited spectrotemporal resolution of the transmitted signal (Başkent, Gaudrain, et al., 2016; Lesica, 2018; Plack, 2018).

While CIs transmit F0 information weakly, still, compared to other voice‐related cues, CI users seem to make more use of F0 cues, albeit to differing degrees for different tasks (Chatterjee & Peng, 2008; Everhardt et al., 2020; Fuller et al., 2014; Gaudrain & Başkent, 2018; Plack, 2018). F0 is considered to be transmitted through HAs due to the amplification of acoustic signal. Nevertheless, as discussed above, the physiological changes related to hearing loss and the typically advanced age of hearing‐impaired individuals, F0 perception mechanisms may be altered internally. Further, in cases of moderate to severe hearing loss, a HA user's individual amplification settings, which are matched to the listeners' hearing loss profile, may limit the range of intensity, alter the shape of the speech spectrum, and temporal envelope, to match the amplification with the impaired hearing physiology, thus potentially delivering pitch contours that differ to that of normal acoustic hearing (Goy et al., 2018; Lesica, 2018). For users of both CIs and HAs, the differing functioning of the auditory perceptual system in tandem with the altered speech signal that the therapeutic device delivers to match the hearing impairment, can influence recognition of vocal emotions. The attenuation of variations in F0 we used to render F0 cues uninformative and the degradation of F0 cues in signals delivered in CI hearing both impact the accuracy with which listeners recognise vocal emotions, e.g., (Luo et al., 2007; Most & Aviner, 2009; Pak & Katz, 2019)—notwithstanding possible confounds of age, with use of assistive listening technologies generally increasing with age (Christensen et al., 2019; Rachman et al., 2022). Consistent with the degradation of the speech signal being less severe in hearing with HAs compared to with CIs, HA users tend to show a milder reduction in accuracy when recognising vocal emotion (Most & Aviner, 2009). While increasing evidence demonstrates that HA and CI listeners have difficulty recognising vocal emotions due to degraded F0 information (e.g., Everhardt et al., 2020; Goy et al., 2018; Most & Aviner, 2009; Waaramaa, Kukkonen, Mykkänen, & Geneid, 2018; Waaramaa, Kukkonen, Stoltz, & Geneid, 2018), little is known about neural mechanisms underpinning poor and successful recognition of vocal emotions in hearing‐impaired individuals.

## 5 CONCLUSIONS

This study found that the ability of normal‐hearing listeners to recognise emotions in speech is impaired by attenuated variations in F0, and that differences in individuals' abilities to do so can be observed in the cortical activations measured from the right STG using fNIRS. Further, this study determined that while fNIRS is not suited to distinguishing between cortical activation evoked by individual emotional categories in speech, it is promising as a non‐invasive method with which to obtain an objective measure of emotion recognition in speech without natural F0 cues.

## AUTHOR CONTRIBUTIONS

Ryssa Moffat: Conceptualization, data curation, formal analysis, investigation, methodology, project administration, visualization, roles/writing ‐ original draft, writing ‐ review and editing. Deniz Başkent: Conceptualization, project administration, resources, supervision, writing ‐ review and editing. Robert Luke: Conceptualization, methodology, software, supervision, visualization, writing ‐ review and editing. David McAlpine: Conceptualization, project administration, resources, supervision, writing ‐ review and editing. Lindsey Van Yper: Conceptualization, methodology, supervision, draft, writing ‐ review and editing.

## CONFLICT OF INTEREST

The authors declare no potential conflict of interest.

## Supporting information


**DATA S1**. Supporting Information

## Data Availability

The data presented in this study are available upon request from the corresponding author, Ryssa Moffat, but are not publicly as participants did not explicitly consent to their data shared being publicly. This type of consent will be requested going forward. All code used in the generation of speech stimuli, as well as the cleaning and analysis of the data described in this study are available at https://osf.io/352qc/.
