# Supplementary material for: Cortical haemodynamic responses predict individual ability to recognise vocal emotions with uninformative pitch cues but do not distinguish different emotions
Source: Hum Brain Mapp. 2023 May 10;44(9):3684–705. doi: 10.1002/hbm.26305 (PMC10203806; doi:10.1002/hbm.26305)
Supplement: Supplementary file 1 — DATA S1. Supporting Information [file HBM-44-3684-s001.pdf]

Supplementary materials: "Cortical haemodynamic responses predict individual ability to recognise vocal emotions with uninformative pitch cues but do not distinguish different emotions"

S1 – Supplementary materials describing stimulus creation for all assessments

Generation of pseudo-sentences

In the pseudo-words *miffox* and *borval*, the final consonant cluster /ks/ in *miffox* does not cause elision of the voiced schwa in *is*, /əz/. The /l/ coda of *borval* is voiced, facilitating the identification of the speech offset for each sentence, which is needed to calculate the speech rate. For the pseudo-words *miffox* and *borval*, six additional rhyming pseudo-words were created by replacing the initial voiced consonant with another voiced consonant, yielding a total of seven sentences (Table S4A).

Item #	Sentence
1	The ziffox is dorval
2	The biffox is jorval
3	The diffox is morval
4	The niffox is zorval
5	The giffox is lorval
6	The miffox is borval
7	The riffox is gorval

Table S1A. Recorded stimulus sentences.

Recording of sentences

During a single recording session in an anechoic chamber, recordings were made using a Røde NT1 microphone with a pop cover (RØDE Microphones, Silverwater, Australia) connected to an RME Fireface UC sound card (RME, Haimhausen, Germany). The sentences were digitally recorded using Adobe Audition (version 13.0.8.43; Adobe Systems, Mountain View, USA) on a personal computer at a sampling rate of 48 kHz (32 bits mono). For each emotion, each of the seven sentences in Table S4A was recorded at least four times. The rendition of each item, for each emotion, with the fewest recording artefacts (i.e., mouth noises created by spittle or lips), mispronunciations and other background noises, was selected and saved as a discrete WAV file using Adobe Audition. Each item was manually trimmed from the onset of the first consonant (initial increase in energy and initial glottal pulse) to the offset of the final consonant (final decrease in energy and final glottal pulse) using Praat (version 6.1.16; Boersma & Weenink, 2018).

Further adjustments

Further adjustments were made to the selection of recordings with the input of lab members and colleagues with experience in the speech and language field in informal pilot listening sessions. First, to ascertain whether the stimuli categories were behaviourally discriminable, 10 experienced listeners were asked to indicate the emotion conveyed by each stimulus in a 4-alternative forced-choice task (7 sentences for each of 4 emotions, N trials=28). During a first round, the response alternatives were labelled as *angry*, *happy*, *sad* or *neutral*. *Happy* and *sad* were commonly misidentified as *neutral* stimuli–consistent with confusions reported by Paulmann et al. (2008) and Scherer et al. (2001). In a second round, 10 new experienced listeners completed the same task, except this time *neutral* was re-labelled *unemotional*. With these alternatives, there were almost no confusions between happy and unemotional (rate of *happy* stimulus being misidentified as *unemotional*=0.01), but the confusions between *sad* and *unemotional* remained (rates for *unemotional* stimulus misidentified as *sad*=0.27, *sad* stimulus mislabelled *unemotional*=0.21). Accuracy was near ceiling in all four emotions (*angry M*=1, *SD*=0; *happy M*=0.94, *SD*=0.1; *sad M*=0.80, *SD*=0.18; *unemotional M*=0.71, *SD*=0.17) indicating that the stimuli are behaviourally discriminable.

Next, to reduce the number of retained recordings, the five sentences with the best expression of each emotion were identified. Eleven new experienced listeners were asked to judge the relative strength with which the relevant emotion was conveyed in each item. All seven sentences for each emotion were presented on a computer screen at once, and listeners were instructed to play each sentence and order them from 'most to least strongly' conveying the relevant emotion. Listeners could play the sentences as often as they wished. The two sentences for each emotion most frequently ordered as 'least strongly' were discarded, yielding sets of five sentences per emotion (Table S4B).

Emotion	Retained sentences	Discarded sentences
angry	1, 2, 4, 5, 6	3, 7
happy	1, 3, 4, 5, 7	2, 6
sad	2, 3, 4, 6, 7	1, 5
unemotional	1, 2, 3, 6, 7	4, 5

Table S1B. Retained sentences for each emotion.

Acoustic measures and acoustic analysis

The mean F0 (Hz), mean intensity (dB, relative to the maximum measured root mean square, RMS), and mean speech rate (syllables per second) were measured for each sentence using a Praat script. To confirm that the emotions are acoustically distinct, i.e., that the emotions differ significantly from one another in at least one acoustic feature, a statistical analysis was performed using R (version 3.6.3; R Core Team, 2020) and the RStudio IDE (version 1.3.959; RStudio Team, 2020). An alpha level of 0.05 was used for all statistical tests. To assess the presence of differences between emotions, a Kruskal-Wallis test was performed for each F0, intensity, and speech rate, revealing significant differences between emotions, i.e., F0 ($\chi^2(3, N=20)=16.07$, $p=0.001$), intensity ($\chi^2(3, N=20)=17.86$, $p<0.001$), and speech rate ($\chi^2(3, N=20)=11.75$, $p=0.008$).

Subsequently, to test for differences between emotions within each acoustic feature (Figure S4A), post-hoc pairwise Wilcoxon rank sum tests were computed with false discovery rate correction for multiple comparisons (FDR; Benjamini & Hochberg, 1995). There is evidence to support significant differences in F0 between the following pairs: mean F0 is higher for each *angry*, *happy* and *sad* relative to *unemotional* (all $W=25$, $p=0.008$), as well as *angry* relative to *sad* ($W=25$, p=0.008), and *happy* relative to *sad* ($W=25$, $p=0.008$). Mean F0 does not differ significantly between *angry* and *happy* ($W=13$, $p=1$). Mean intensity differs significantly between all pairs (all $W=25$, $p=0.008$). Mean speech rate is significantly higher for *happy* than each *angry*, *unemotional*, and *sad* (all $W=25$, $p=0.008$). Mean speech rate does not differ significantly between *angry* and *unemotional* ($W=6$, $p=0.222$), *angry* and *sad* ($W=8$, $p=0.421$), or *sad* and *unemotional* ($W=13$, $p=1$).

Despite the small number of stimuli and variability within each emotion, each emotion patterns in an acoustically discriminable fashion. The mean values and the relative positions of emotions within each acoustic feature match those previously reported for F0 and rate (Paulmann & Uskul, 2014); for F0 (Gilbers et al., 2015; Pell, 1998), for intensity (Luo et al., 2007), for rate (Most & Aviner, 2009). The direction of differences between emotions is also similar to those previously described for each acoustic feature (Belin et al., 2008; Murray & Arnott, 1993; Paulmann & Kotz, 2008; Pollermann & Archinard, 2002; Yildirim et al., 2004)

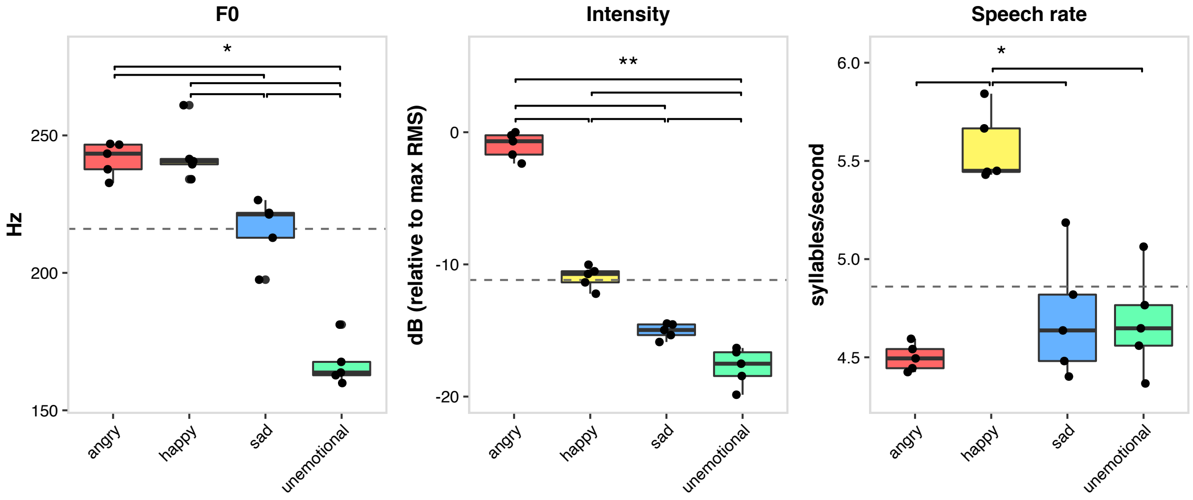

Figure S1A*.* Analyses of acoustic feature within each emotion. The dashed line indicates the overall mean across emotions. Significance for Wilcoxon rank sum test, *$p<0.05$; **$p<0.01$.

Creation of conditions
Next, through the creation of five speech conditions, F0, intensity, and speech-rate cues were systematically rendered uninformative by attenuating variations in the given feature. The five conditions were named according to the attenuated feature(s): a) *natural* (all variations in features intact), b) *intensity+rate*, c) *F0*, d) *intensity+F0*, e) *rate+F0*. In the natural condition, the signal was not altered, thus leaving variations in investigated acoustic features intact. To create the *intensity+rate*, *F0*, *intensity+F0*, and *rate+F0* conditions, variations in the investigated features were attenuated sequentially. Variations in F0 were attenuated first, followed by variations in intensity, then speech rate (e.g., for the *rate+F0* condition, variations in F0 were attenuated before variations in speech rate; features names were added to condition name as prefixes as they were subjected to attenuation of variations). Next, the speech stimuli were prepared for each assessment type (i.e., individual sentences for behavioural assessments, 5-sentence blocks of each emotion-condition pair for fNIRS sessions) and matched to presentation sound level (Figure S4B).

The following section will describe the signal processing procedures applied to the speech signal to attenuate variations in each F0, intensity and speech rate, as well as the procedures used to prepare speech for presentation in behavioural and fNIRS assessments (Figure S4B). After attenuating variations in a cue, the acoustic properties of the stimuli were measured to ensure that the selected signal processing procedure did attenuate variations to the target value (Figure 2.4). The process of attenuating variations in any cue can alter other aspects of the speech signal. This is unavoidable, and where possible, measures were taken to minimise these alterations. The procedures, their success, and any observed influence on other untargeted acoustic features or the signal in general will be described next.

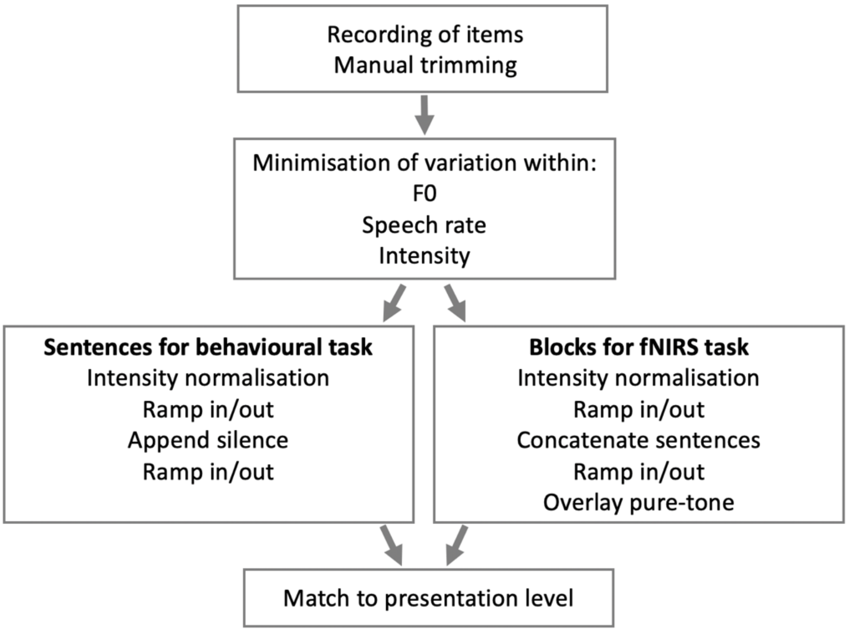

Figure S1B. Stimulus creation pipeline. Flowchart illustrating the order of steps taken to create single-sentence stimuli for behavioural assessments and 5-sentence blocks fNIRS sessions.

Attenuation of F0 variation
Variations in F0 were attenuated using the PyWORLD vocoder python package(version 0.2.11; Morise et al., 2016). First, the F0 contour is extracted using Distributed Inline-filter Operation algorithm (DIO; Drugman et al., 2018). The F0 floor and ceiling were set to 100 and 800 Hz, respectively. The estimated F0 contour was then refined using StoneMask (Al-Radhi et al., 2019)— an algorithm that reduces the effects of noise on the estimated F0 values. Second, the spectral envelope was extracted using CheapTrick (Morise, 2015) with its default parameters (a spectral recovery parameter of -0.15 and automatically computed FFT size that allows accurate representation of F0 floor). Third, the aperiodicity was estimated using Definitive Decomposition Derived Dirt-Cheap (d4c; Morise, 2016). The threshold for the aperiodicity-based voiced/unvoiced decision was set to the default 0.85. Finally, the original F0

contour was replaced with a constant F0 of 217 Hz—the mean F0 across all stimuli—and the speech signal was reconstructed based on the target F0, spectrogram, and aperiodicity.

As displayed under the experimental condition *F0* in Figure S4C, manipulating F0 may have a small influence on the RMS of *angry* stimuli; the RMS mean, median and variability are slightly increased for *angry* in the *F0* condition. Attenuating variations in F0 does not appear to have influenced speech rate or introduced any obvious abnormalities to the general signal.

Attenuation of intensity variation
Variations in intensity were attenuated using a Praat 'intensity-neutralizer' script shared by the UCLA (University of California, Los Angeles) Phonetics Lab (Vicenik, n.d.-a). To attenuate variations in intensity to the target value across a WAV file (here: -11.19 dB relative to max RMS—the mean across all natural stimuli), the function measured the intensity of the signal at 10-ms-intervals. At each interval, the difference between the measured and target intensity was obtained. Next, at each interval, the intensity was scaled to the target intensity of -11.19 dB (relative to max RMS).

Attenuating variations in intensity caused a slight reduction of the mean F0 of *angry* stimuli, as illustrated under the intermediate condition *Intensity* in Figure S4C. It did not influence speech rate. One drawback of the applied procedure is that it flattened the intensity of all speech segments and periods of silence, with the latter resulting in periods of white noise (illustrated in Figures S4D and S4E, *intensity+rate* and *intensitity+F0*). In 25% of the *intensity+rate* stimuli and 35% of the *intensity+F0* stimuli, attenuating variations in intensity introduced a 20 to 30-ms click between the first two words in the sentence. Attempts to resolve this issue failed to systematically reduce the unwanted noise without influencing the overlap between words. Rather than further distorting the speech signal, clicks were accounted for as a difference between stimulus sentences by including 'sentence' as a random effect when using generalised mixed-effects models (GLMMs) to predict the accuracy of vocal emotion recognition. Note that the conditions containing stimuli with clicks were not included in fNIRS sessions.

Attenuation of speech rate variation
Variations in speech rate were attenuated at the sentence level using the audioTSM python package (Muges, 2017). The phase-vocoder function was used to adjust the speed of the speech by a scaling factor (equal to the original duration divided by the target duration). This function implements the Waveform Similarity-based Overlap-Add procedure (WSOLA; Verhelst & Roelands, 1993). WSOLA uses the overlap-add (OLA) procedure in which the signal is windowed, and the windows are concatenated with a fixed amount of overlap, which lengthens or shortens the signal. The 'waveform similarity' is an additional procedure that allows a variable, instead of fixed, amount of overlap, depending on the similarity of the concatenated windows, to preserve periodic signal components, i.e., reduce aperiodic artefacts. The target duration was set to 1.24 s, which is equal to 4.82 syl/s. The duration of the resulting WAV files was *M*=1.22 s (*SD*=0 s), which is equal to 4.92 syl/s. This duration is slightly shorter than the target duration. This deviation from the target, as well as the minimal variability between files (~0.004 s), can be attributed to the precision of the WSOLA procedure.
Attenuating variations in speech rate resulted in a reduction of the RMS for all emotions, as evidenced under the intermediate condition *rate* in Figure S4C. There was also a small increase in mean F0 for all emotions except *happy*, mirroring the relative change in speech rate (i.e., increased for all emotions except *happy*).

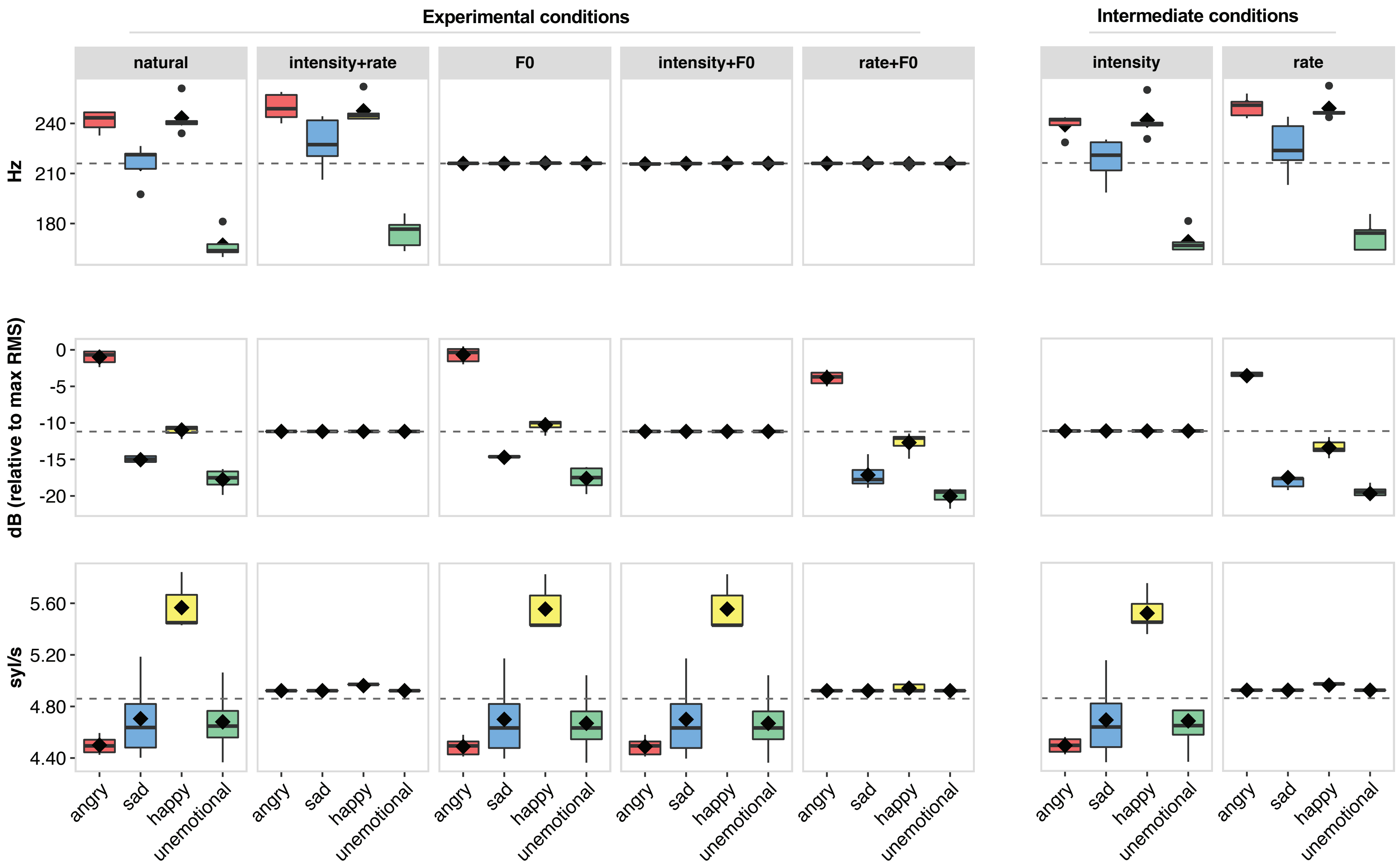

Experimental conditions
Intermediate conditions
natural
intensity+rate
F0
intensity+F0
rate+F0
intensity
rate
Hz
240
210
180
dB (relative to max RMS)
0
-5
-10
-15
-20
syl/s
5.60
5.20
4.80
4.40
angry
sad
happy
unemotional

Figure S1C. Measured acoustic properties for each emotion within each experimental condition, and two intermediate conditions. The first row shows mean F0 in Hz, the second shows mean intensity in dB relative to the max RMS, the third shows mean speech rate in syl/s. The columns indicate how the signal has been changed. The columns for experimental conditions illustrate the acoustic properties of the experimental stimuli and the supplementary columns for intermediate conditions illustrate the acoustic properties of conditions that were not included in the assessments (i.e., *intensity* and *rate* alone). The intermediate conditions provide insight into the alterations of the speech signal incurred by attenuation of variations within those features. Where variations in a feature were not attenuated (i.e., F0 in the *intensity+rate* condition), the distribution of the emotions resembles the *natural* condition. Where variations in a feature have been attenuated (i.e., F0 in *F0* condition), all emotions have the same mean value as the target value (dashed line; mean across all emotions).

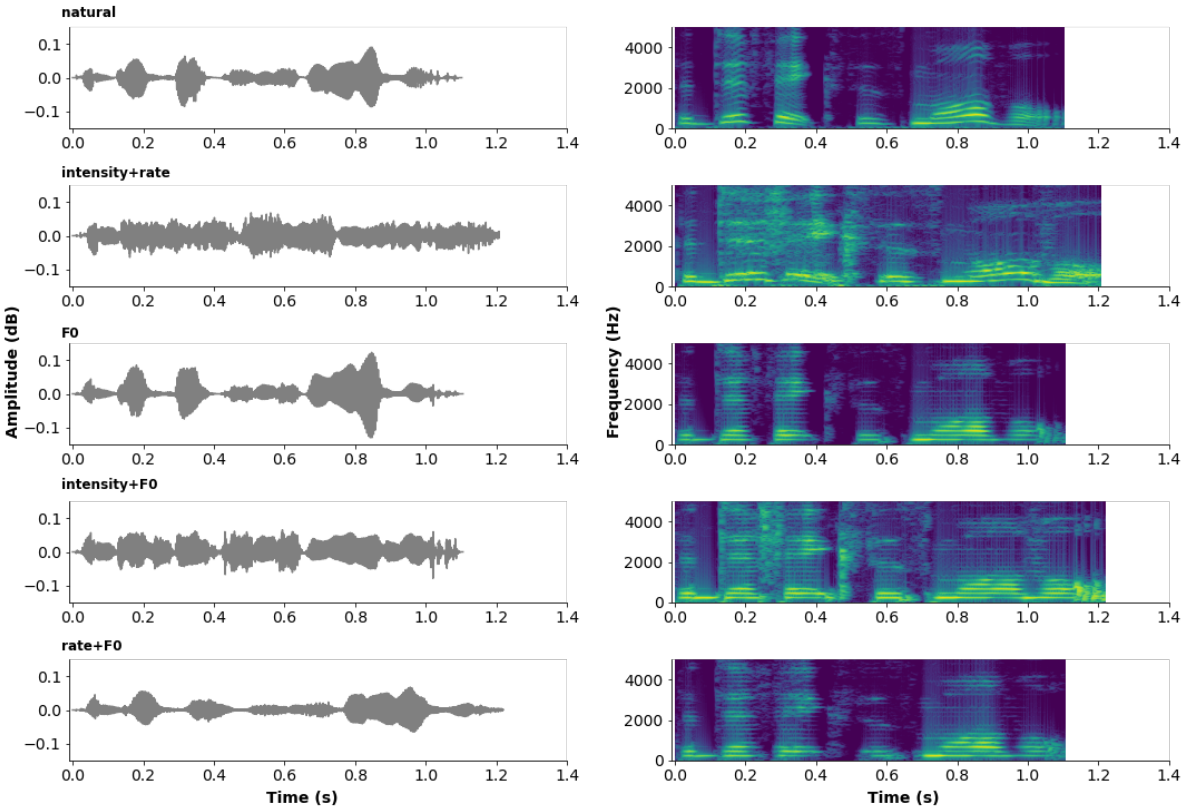

Figure S4D. *Happy* (sentence 3), waveforms and narrowband spectrograms per condition.

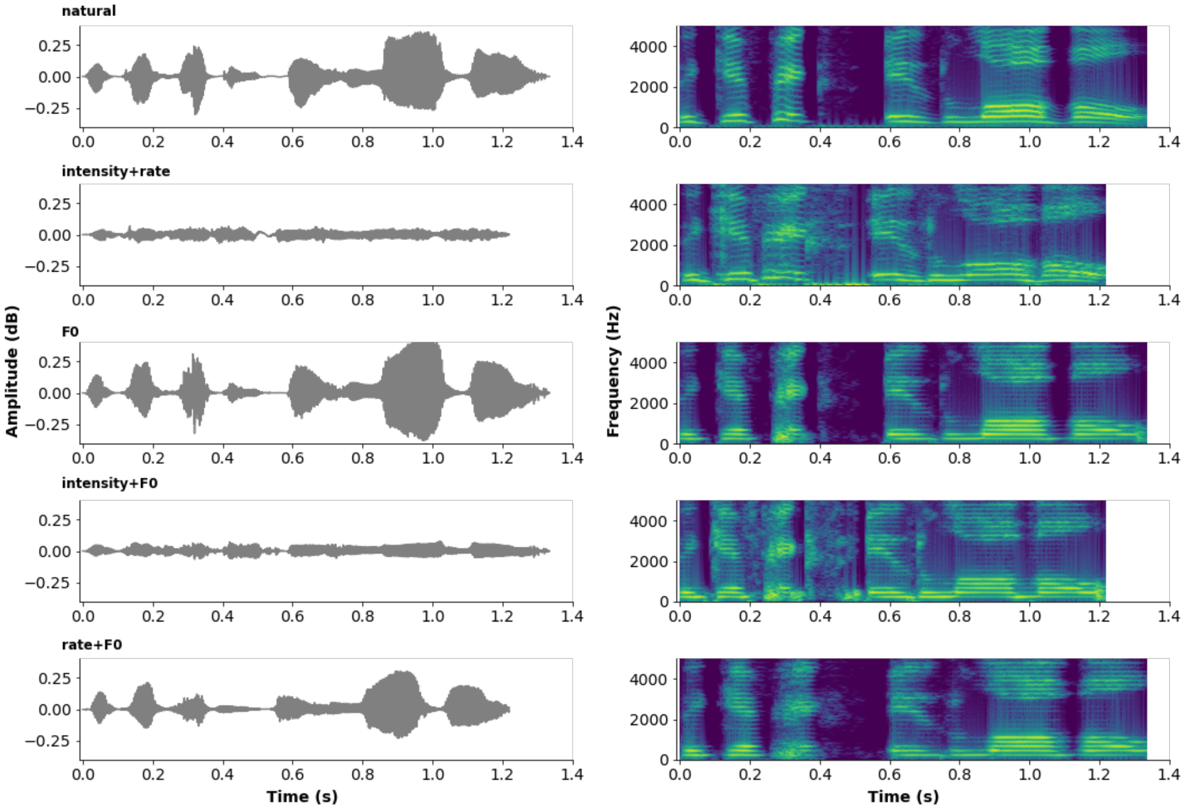

Figure S1E. *Angry* (sentence 5), waveforms and narrowband spectrograms per condition.

Preparing stimuli for behavioural and fNIRS assessments

Intensity normalisation

With the exception of the sentence-length stimuli presented in the first behavioural assessment, where intensity was investigated as a cue for emotion recognition, the overall intensity of all stimuli was normalised (fNIRS sessions 1 and 2). To ensure that the overall intensity of all stimuli was equal, a UCLA Phonetics Lab Praat 'intensity-scaler' script (Vicenik, n.d.-b) was adapted for this purpose. Using this script, the signal of each WAV file was multiplied by a scaling factor to obtain an overall intensity of -11.19 dB (relative to max RMS). Subsequently, a 10-ms ramp was applied to beginning and end of each sentence stimuli using the Pysox python package (version 1.4.0; Bittner et al., 2016). This served to smooth onset and offsets of speech, alleviating clicks.

Blocks for fNIRS task

Using the intensity-normalised and ramped stimuli, a block was created for each emotion in each of the *natural* and *F0* conditions. Blocks were generated by concatenating the five sentences for each emotion with the Pysox package. The sentences within a block were separated by 200 ms of silence and bookended with 100 ms of silence. Another 10-ms ramp was applied to the beginning and end of the WAV file for each block using the Pysox package.

For each fNIRS session, five additional stimuli were created to be used in attention-keeping trials (i.e., trials eliciting a button-press response upon hearing a tone to ensure the participant was attending to the stimuli). These stimuli were identical to the experimental stimuli, with an additional pure tone overlapping with the speech at a random time. To create these, each of the experimental stimuli was copied and a 500-ms-long 400-Hz pure tone (mean intensity of -18.34 dB relative to maximum stimulus RMS) generated in Praat, was overlaid on the speech using Adobe Audition. The tone's intensity was selected to ensure that the attention task was sufficiently challenging to maintain participants' attention but that the tone was not overly salient or shocking.

Following the intensity normalisation and ramping, an additional 200 ms of silence was appended to the beginning and 100 ms to the end of all sentence stimuli using the Pysox package. This was done to ensure that the stimuli would not be interrupted by buffering delays. Next, 10-ms ramps were applied to the beginning and end of the WAV files.

Matching to presentation level

In a final step, all behavioural and fNIRS stimuli were matched to the target presentation level. For the first behavioural assessment on vocal emotion recognition, the target level was 60 dBA. Participants reported that subjectively, the stimuli were presented at a comfortable level, but could be presented at a higher level and still be comfortable. As stimulus intensity is positively correlated to the amplitude of auditory haemodynamic responses (Weder et al., 2018), which are intrinsically relatively small (e.g., relative to motor responses), the target level was increased to 70 dBA for both fNIRS sessions and the associated behavioural assessments, to ensure that reliable haemodynamic responses could be obtained. Sound-level matching was achieved using a white noise generated in Praat, which was played in Presentation through an RME Fireface UC attached to Etymotic ER2 (first behavioural assessment) or ER3 (fNIRS and behavioural re-assessment) insert phones (Etymotic Research, Inc., Elk Grove Village, USA). The long-term average sound pressure level (in dBA) of the white noise was measured over ~20 s using a 2-cc ear simulator (RA0045, G.R.A.S., Twinsburg, USA) and a B&K Type 2250 sound level meter (Brüel & Kjær, Nærum, Denmark). The measured level (96 dBA) was used to calculate the RMS of the white noise, which served as the reference RMS. Using this reference, the RMS was calculated for a calibration track consisting of concatenated *intensity+rate* sentences with no silences (the silence threshold was set to -40 dB). Using these RMS values and the reference RMS, the calibration track and the stimuli were then scaled to the target presentation level (i.e., 60 dBA for the first behavioural assessment, and 70 dBA for both fNIRS sessions and associated behavioural assessments).

Final stimuli for behavioural and fNIRS assessments

In the first behavioural assessment, the experimental paradigm comprised *angry*, *happy*, *sad*, and *unemotional* sentences, presented in five conditions (*natural, intensity+rate*, *F0*, *intensity+F0*, *rate+F0*). In both fNIRS sessions, a reduced number of conditions and/or emotions was presented, with a silent control condition and 10 attention trials, to respect the time constraints imposed by fNIRS recordings. For the first fNIRS session, the block-design paradigm included each *angry*, *happy*, *sad*, and *unemotional* trials (*natural* condition only), the silent control trials, as well as attention and practice trials

(total trials=114, duration=50 minutes). In the second fNIRS session, the block-design paradigm included two emotions (*happy* and *sad*), in two speech conditions (*natural* and *F0*), as well as the silent control, attention, and practice trials (total trials=144, duration=50 minutes).

***S2* – Supplementary materials for behavioural assessment of emotion recognition**

All emotions	**Model 1:** Accuracy ~ Contrast1 + Contrast2 + Contrast3 + Contrast4 + (1+ Contrast2||Participant) + (1|Sentence) + (1 + Contrast2 + Contrast3 + Contrast4 ||Emotion)				
	$R^2_{m/c}$ = 0.38/0.55				
	β	***SE***	***CI***	**z**	***p***
Intercept	1.22	0.49	0.00, 0.64	2.51	0.012
Contrast1	0.72	0.19	0.34, 1.12	3.73	<0.001
Contrast2	2.84	0.85	0.73, 4.98	3.33	0.001
Contrast3	0.47	0.47	-0.79, 1.62	0.88	0.381
Contrast4	0.45	0.45	-1.51, 0.79	-0.80	0.424

Angry	**Model 2:** Accuracy ~ Contrast1 + Contrast2 + Contrast3 + Contrast4 + (1 + Contrast2||Participant)				
	$R^2_{m/c}$ = 0.93/0.95				
	β	***SE***	***CI***	**z**	***p***
Intercept	5.98	16.78	3.22, Inf	0.36	0.721
Contrast1	15.69	83.88	0.85, Inf	0.19	0.852
Contrast2	3.10	0.79	1.72, 4.92	3.90	<0.001
Contrast3	2.46	0.30	1.90, 3.06	8.31	<0.001
Contrast4	-2.24	0.29	-2.82, -1.70	-7.86	<0.001

Happy	**Model 3:** Accuracy ~ Contrast1 + Contrast2 + Contrast3 + Contrast4 + (1 + Contrast2||Participant) +(1|Sentence)				
	$R^2_{m/c}$ = 0.63/0.81				
	β	***SE***	***CI***	**z**	***p***
Intercept	0.16	0.38	-0.63, 0.96	0.41	0.680
Contrast1	0.85	0.38	-0.06, 1.82	0.41	0.680
Contrast2	6.08	0.68	4.90, 7.69	8.90	<0.001
Contrast3	0.10	0.32	-0.52, 0.72	0.32	0.752
Contrast4	0.44	0.33	-0.21, 1.10	1.31	0.188

Sad	**Model 4:** Accuracy ~ Contrast1 + Contrast2 + Contrast3 + Contrast4 + (1 + Contrast2||Participant) + (1|Sentence)				
	$R^2_{m/c}$ = 0.56/0.67				
	β	***SE***	***CI***	**z**	***p***
Intercept	0.65	0.34	-0.11, 1.43	1.92	0.055
Contrast1	0.19	0.32	-0.66, 1.05	0.43	0.666
Contrast2	4.73	0.52	3.77, 5.86	9.01	<0.001
Contrast3	0.27	0.26	-0.24, 0.79	1.04	0.299
Contrast4	-0.61	0.26	-1.12, -0.11	-2.39	0.017

Unemotional	**Model 5:** Accuracy ~ Contrast1 + Contrast2 + Contrast3 + Contrast4 + (1+ Contrast2||Participant) +(1|Sentence)				
	$R^2_{m/c}$ = 0.04/0.42				
	β	***SE***	***CI***	**z**	***p***
Intercept	1.84	0.34	1.12, 2.61	5.34	<0.001
Contrast1	1.10	0.29	0.51, 1.73	3.71	<0.001
Contrast2	-0.01	0.37	-0.75, 1.73	-0.01	0.990
Contrast3	-0.85	0.28	-1.44, -0.29	-3.01	0.003
Contrast4	0.78	0.28	0.21,1.36	2.75	0.006

Table S2A. Model coefficients for sliding difference contrasts in models fit to first behavioural assessment. Contrasts compared between consecutive condition, which were ordered from hypothesised best-to-most impaired emotion recognition. Contrast1=*natural* vs. *intensity+rate*, Contrast2= *intensity+rate* vs. *F0*, Contrast3= *F0* vs. *F0+intensity*, Contrast4=*F0+intensity* vs. *F0+rate*. For *angry* speech, the confidence intervals for the intercept and Contrast1 include Inf, as a result of the ceiling effects observed in the *natural* and *Intensity+Rate* conditions. Despite this, these contrasts are presented for consistency across the emotions. Neither is estimate is statistically significant.

S3 – Supplementary materials for fNIRS session 1 (*angry*, *happy*, *sad*, and *unemotional* in natural speech)

Generation of grand average waveforms (same for sessions 1 and 2)
First, grand average waveforms were generated for visual inspection of the fNIRS data. The data were resampled to 3 Hz, and raw intensity was converted to optical density. As a measure of signal quality, a scalp-coupling index (Pollonini et al., 2014) was calculated per channel for frequencies between 0.7-1.35 Hz. 93% and 94% of channels had a scalp-coupling index >0.8 in fNIRS sessions 1 and 2 respectively, indicating good contact between optodes and the scalp (Figures S2A and S3A). Channels with scalp-coupling-index (SCI) values <0.8 were rejected. Motion artefacts were corrected using the temporal derivative distribution repair (TDDR) algorithm (Fishburn et al., 2019). Next, short-channel regression was applied using the nearest short channel for each long channel, effectively isolating the cerebral signal component by regressing out extracerebral and systemic components (Saager & Berger, 2005; Scholkmann et al., 2014). The signal was then converted from optical density to concentrations of HbO and HbR using the Modified Beer-Lambert Law (Delpy et al., 1988; Kocsis et al., 2006) with a partial pathlength factor of 0.1. Next, we applied Cui et al.'s (2010) algorithm to improve signal-to-noise ratio based on the negatively correlated dynamics of HbO and HbR. The signal was then bandpass-filtered between 0.01–0.4 Hz to exclude slow drifts and cardiac components. Response epochs were trimmed from 5 s before stimulus onset to 20 s post-onset and linearly detrended, accounting for slow drifts. Epochs with peak-to-peak differences >200 μM were excluded from the qualitative analysis.

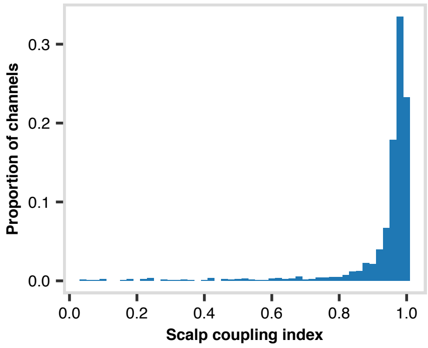

Figure S3A. Signal quality as measured by scalp-coupling index per channel for the first fNIRS session; histogram showing the distribution of scalp-coupling indices, calculated per channel, as a proportion of the total number of channels (N=2856).

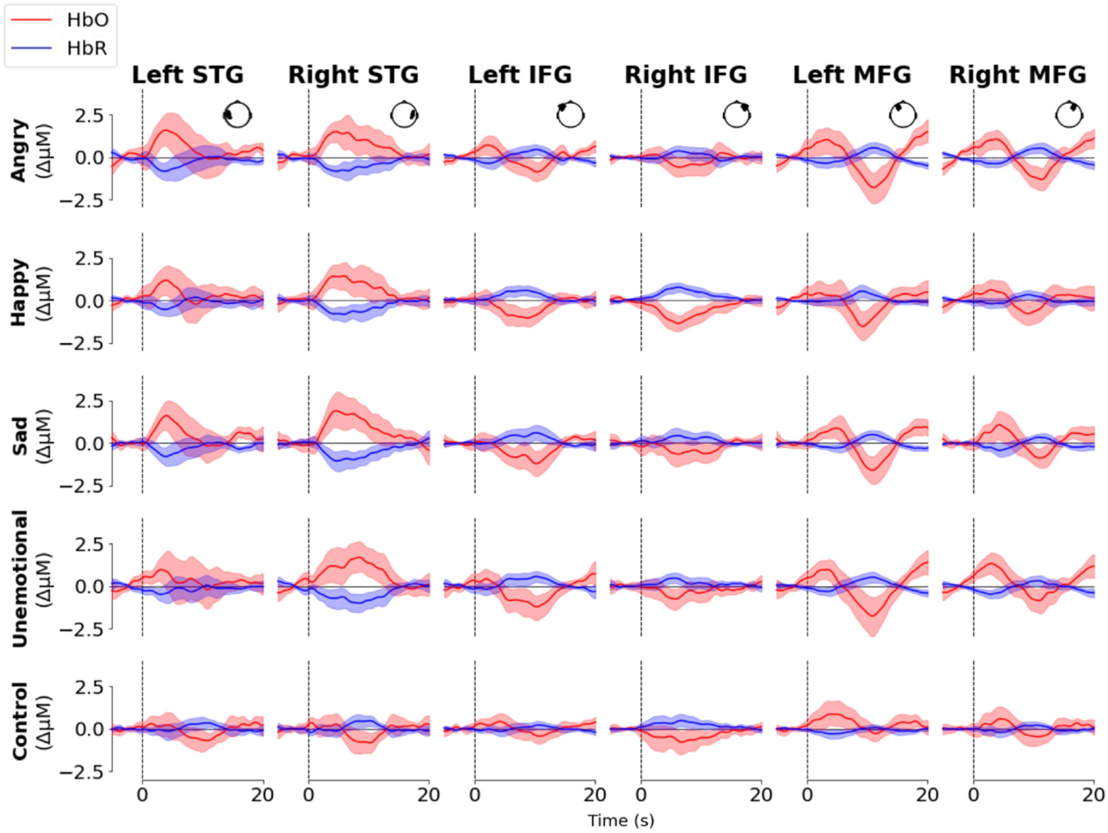

Figure S3B. Waveforms including MFG from fNIRS session 1 (emotions in natural speech only).

		HbO					HbR				
		**Model 6:** β ~ -1 + ROI + Condition + ROI:Condition + (1 + Condition|Participant)					**Model 7:** β ~ -1 + ROI + Condition + ROI:Condition + (1 + Condition|Participant)				
		$R^2_{m/c}$ = 0.15/0.45					$R^2_{m/c}$ = 0.31/0.53				
	**ROI**	**β**	***SE***	***CI***	***t***	***p***	**β**	***SE***	***CI***	***t***	***p***
**Angry**	LSTG	**2.49**	**0.64**	**1.22, 3.77**	**3.90**	**<0.001**	**-1.70**	**0.28**	**-2.27, -1.13**	**-5.98**	**<0.001**
	RSTG	**3.25**	**0.64**	**1.98, 4.52**	**5.07**	**<0.001**	**-1.90**	**0.28**	**-2.47, -1.33**	**-6.68**	**<0.001**
	LIFG	0.20	0.64	-1.07, 1.47	0.32	0.753	0.22	0.28	-0.35, 0.79	0.78	0.439
	RIFG	0.13	0.64	-1.15, 1.40	0.20	0.844	0.14	0.28	-0.43, 0.71	0.49	0.628
	LMFG	-0.19	0.64	-1.47, 1.07	-0.31	0.758	-0.02	0.28	-0.55, 0.59	-0.07	0.947
	RMFG	0.48	0.64	-0.79, 1.75	0.75	0.456	-0.20	0.28	-0.776, 0.37	-0.69	0.492
**Happy**	LSTG	**1.99**	**0.66**	**0.69, 3.29**	**3.05**	**0.003**	**-0.97**	**0.27**	**-1.51, -0.42**	**-3.52**	**0.001**
	RSTG	**2.72**	**0.65**	**1.42, 4.02**	**4.16**	**<0.001**	**-1.94**	**0.27**	**-2.47, -1.40**	**-7.18**	**<0.001**
	LIFG	0.10	0.65	-1.20, 1.41	0.16	0.875	0.22	0.27	-0.33, 0.76	0.78	0.435
	RIFG	-0.25	0.67	-1.58, 1.07	-0.38	0.703	0.28	0.27	-0.26, 0.81	1.02	0.310
	LMFG	-0.51	0.65	-1.81, 0.80	-0.77	0.441	0.14	0.27	-0.39, 0.68	0.54	0.593
	RMFG	-0.28	0.65	-1.58, 1.02	-0.43	0.668	-0.08	0.27	-0.62, 0.45	-0.31	0.756
**Sad**	LSTG	**1.67**	**0.66**	**0.35, 2.99**	**2.53**	**0.014**	**-1.30**	**0.25**	**-1.80, -0.80**	**-5.13**	**<0.001**
	RSTG	**2.80**	**0.66**	**1.48, 4.11**	**4.22**	**<0.001**	**-1.89**	**0.25**	**-2.39, -1.39**	**-7.48**	**<0.001**
	LIFG	**-1.71**	**0.67**	**-3.05, -0.37**	**-2.54**	**0.013**	0.09	0.25	-0.42, 0.59	0.34	0.736
	RIFG	0.06	0.66	-1.26, 1.38	0.09	0.931	-0.01	0.25	-0.51, 0.49	-0.03	0.975
	LMFG	-0.42	0.66	-1.74, 0.90	-0.63	0.529	0.03	0.25	-0.47, 0.53	0.13	0.894
	RMFG	-0.39	0.66	-1.71, 0.93	-0.59	0.559	-0.30	0.25	-0.80, 0.20	-1.20	0.233
**Unemotional**	LSTG	**1.42**	**0.67**	**0.08, 2.76**	**2.11**	**0.038**	**-1.51**	**0.24**	**-1.99, -1.03**	**-6.28**	**<0.001**
	RSTG	**2.60**	**0.67**	**1.25, 3.94**	**3.85**	**<0.001**	**-1.94**	**0.24**	**-2.41, -1.46**	**-8.05**	**<0.001**
	LIFG	-0.79	0.67	-2.13, 0.56	-1.17	0.247	0.16	0.24	-0.31, 0.64	0.68	0.499
	RIFG	-0.41	0.67	-1.76, 0.93	-0.62	0.540	0.03	0.24	-0.45, 0.50	0.11	0.914
	LMFG	0.19	0.67	-1.15, 1.53	0.28	0.779	0.13	0.24	-0.35, 0.60	0.52	0.603
	RMFG	0.52	0.67	-0.82, 1.87	0.78	0.439	-0.09	0.24	-0.56, 0.39	-0.36	0.717
**Control**	LSTG	-0.28	0.57	-1.40, 0.84	-0.50	0.622	0.30	0.25	-0.19, 0.80	1.21	0.228
	RSTG	-0.58	0.57	-1.70, 0.54	-1.02	0.310	0.30	0.25	-0.19, 0.79	1.21	0.230
	LIFG	0.18	0.57	-0.94, 1.30	0.32	0.752	0.09	0.25	-0.40, 0.58	0.36	0.720
	RIFG	0.33	0.57	-0.79, 1.45	0.58	0.561	0.40	0.25	-0.09, 0.89	1.60	0.111
	LMFG	0.03	0.57	-1.08, 1.15	0.06	0.954	0.00	0.25	-0.49, 0.49	-0.01	0.996
	RMFG	0.48	0.57	-0.64, 1.60	0.85	0.398	0.07	0.25	-0.42, 0.57	0.30	0.767

Table S3B. Group-level estimates of haemodynamic response amplitude. Significance of contrasts, bold font indicates p<0.05

Results of channel-wise analysis
To assess haemodynamic response amplitude on a per-channel basis, we fit the following model: β ~ -1 + Channel:Condition + (1 + Condition|Participant). The estimates are visualised in Figure S3C. Haemodynamic response with increased HbO and decreased HbR are well distributed across bilateral STG only, with the exception of the same pattern observed for angry in channel S20_D10, which was not assigned to an ROI, but can be ascribed to the MFG, albeit with reduced specificity.

Next, we contrasted each emotion against *unemotional* using FDR correction for multiple comparisons, and once with no correction for multiple comparisons to provide additional context for the interpretation of these results. All channels showing significant activity in a minimum of one speech condition were included in the contrasts. Results of contrast are reported in Table S3C for HbO and Table SCD below for HbR. With FDR correction for multiple comparisons, no channels showed any change in activation for either HbO or HbR. Without correction for multiple comparisons, *angry* evoked increased HbO and HbR than unemotional in channel S5_D3, in left IFG, and *happy* evoked increased HbO and HbR in channels S6_D2 (left IFG) and S19_D10 (right IFG). Increased HbR was also observed in uncorrected contrasts in channel S10_D4 (anterior STG or posterior IFG, left) for *happy*.

Interpretation of channel-wise analysis
Based on the high number of comparisons and the simultaneous increase in concentrations of both chromophores (as opposed to negatively correlated HbO and HbR), we interpret the uncorrected results with extreme caution. The channels identified as showing significantly greater HbO and HbR amplitudes for a given emotion relative to unemotional were only showed partial activation to one single speech condition. In other words, when we verified whether response amplitude differed significantly from zero per channel and per condition, channels S5_D3, S6_D2, and S19_D10 only showed significant activation indexed by HbR, not HbO, each for a single speech condition (Figure S3C). This may reflect some affective processing, albeit very weakly, but is more likely to reflect physiological haemodynamic changes (e.g., in blood pressure), as the estimates for HbO and HbR are both above zero (Figure S3C). As a result, we posit that the channel-wise analysis yields only underwhelming evidence for unique haemodynamic profiles of any individual, and that this alternative analysis thereby corroborates our findings in our primary analysis of the first fNIRS assessment.

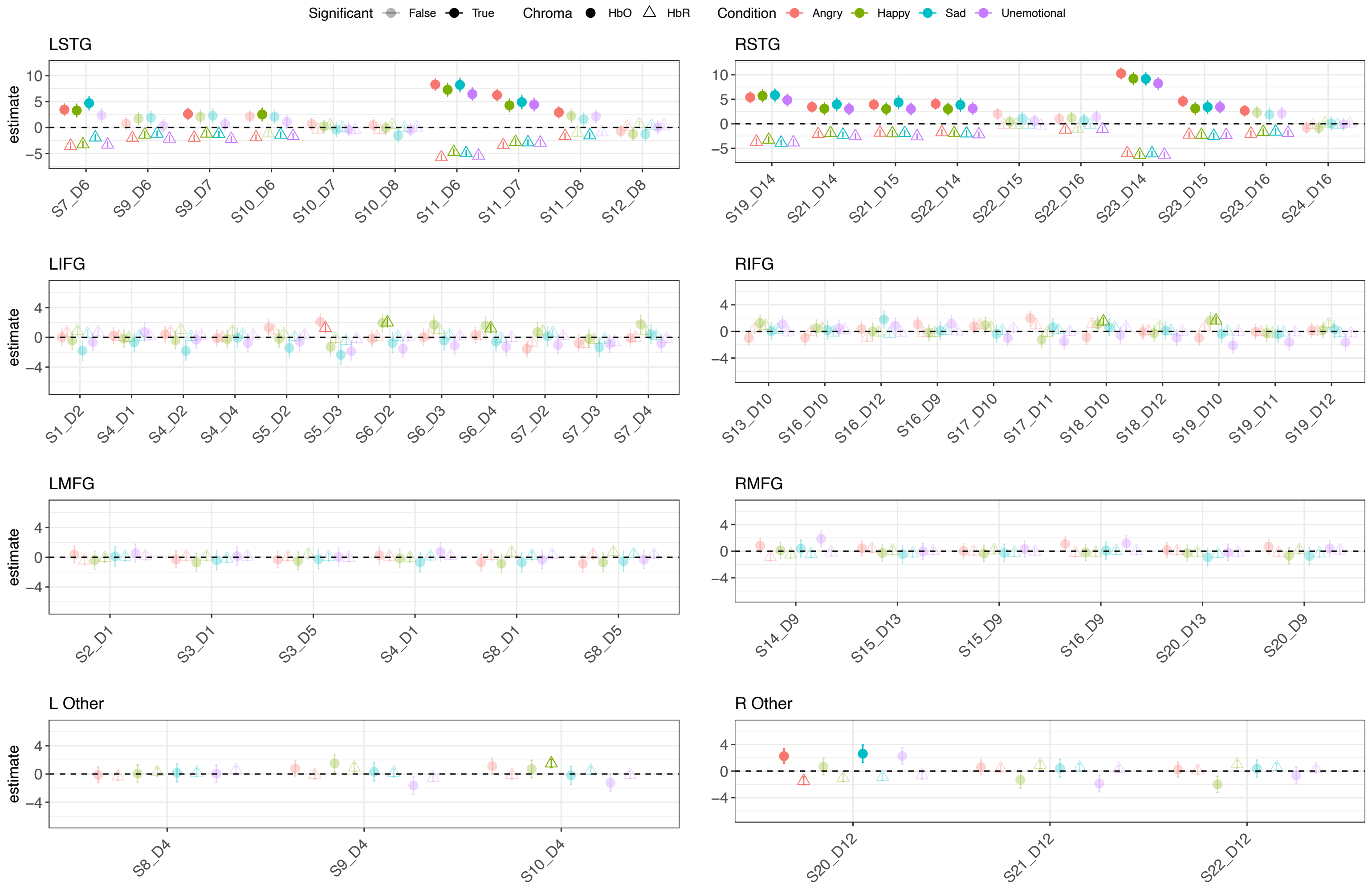

Figure S3C. Estimate per channel from channel-wise second level analysis. Bars show standard error. Transparent symbols indicate *p*>0.05, filled symbols indicate $p < 0.05$.

Table S3C. HbO: Contrast between each emotion and *unemotional* for channel in which speech evoked at least one significant haemodynamic response (HbO or HbR for any condition). Significance of contrasts, bold font indicates p<0.05.

	FDR corrected				Uncorrected			
**Contrast**	**Estimate**	**SE**	**z**	**p**	**Estimate**	**SE**	**z**	**p**
S7_D6 A-U	1.08	1.65	0.65	0.97	1.08	1.65	0.65	0.51
S7_D6 H-U	0.93	1.75	0.53	0.97	0.93	1.75	0.53	0.60
S7_D6 S-U	2.34	1.47	1.59	0.97	2.34	1.47	1.59	0.11
S9_D6 A-U	0.43	1.65	0.26	0.98	0.43	1.65	0.26	0.80
S9_D6 H-U	1.41	1.75	0.81	0.97	1.41	1.75	0.81	0.42
S9_D6 S-U	1.58	1.47	1.07	0.97	1.58	1.47	1.07	0.28
S9_D7 A-U	1.92	1.65	1.16	0.97	1.92	1.65	1.16	0.24
S9_D7 H-U	1.40	1.75	0.80	0.97	1.40	1.75	0.80	0.43
S9_D7 S-U	1.59	1.47	1.08	0.97	1.59	1.47	1.08	0.28
S10_D6 A-U	1.03	1.65	0.62	0.97	1.03	1.65	0.62	0.53
S10_D6 H-U	1.42	1.75	0.81	0.97	1.42	1.75	0.81	0.42
S10_D6 S-U	0.97	1.47	0.66	0.97	0.97	1.47	0.66	0.51
S11_D6 A-U	1.84	1.65	1.12	0.97	1.84	1.65	1.12	0.26
S11_D6 H-U	0.83	1.75	0.47	0.97	0.83	1.75	0.47	0.64
S11_D6 S-U	1.76	1.47	1.20	0.97	1.76	1.47	1.20	0.23
S11_D7 A-U	1.83	1.65	1.11	0.97	1.83	1.65	1.11	0.27
S11_D7 H-U	0.47	1.47	0.32	0.97	0.47	1.47	0.32	0.75
S11_D7 S-U	0.47	1.47	0.32	0.97	0.47	1.47	0.32	0.75
S11_D8 A-U	0.80	1.65	0.49	0.97	0.80	1.65	0.49	0.63
S11_D8 H-U	0.12	1.75	0.07	1.00	0.12	1.75	0.07	0.95
S11_D8 S-U	-0.55	1.47	-0.37	0.97	-0.55	1.47	-0.37	0.71
S19_D14 A-U	0.55	1.65	0.34	0.97	0.55	1.65	0.34	0.74
S19_D14 H-U	0.85	1.75	0.49	0.97	0.85	1.75	0.49	0.63
S19_D14 S-U	1.01	1.47	0.68	0.97	1.01	1.47	0.68	0.50
S21_D14 A-U	0.40	1.65	0.24	0.98	0.40	1.65	0.24	0.81
S21_D14 H-U	0.05	1.75	0.03	1.00	0.05	1.75	0.03	0.98
S21_D14 S-U	0.91	1.47	0.62	0.97	0.91	1.47	0.62	0.54
S21_D15 A-U	0.90	1.65	0.54	0.97	0.90	1.65	0.54	0.59
S21_D15 H-U	-0.01	1.75	0.00	1.00	-0.01	1.75	0.00	1.00
S21_D15 S-U	1.35	1.47	0.91	0.97	1.35	1.47	0.91	0.36
S22_D14 A-U	0.95	1.65	0.58	0.97	0.95	1.65	0.58	0.56
S22_D14 H-U	-0.11	1.75	-0.06	1.00	-0.11	1.75	-0.06	0.95
S22_D14 S-U	0.75	1.47	0.51	0.97	0.75	1.47	0.51	0.61
S22_D16 A-U	-0.37	1.65	-0.22	0.98	-0.37	1.65	-0.22	0.82
S22_D16 H-U	-0.20	1.75	-0.12	1.00	-0.20	1.75	-0.12	0.91
S22_D16 S-U	-0.68	1.47	-0.46	0.97	-0.68	1.47	-0.46	0.64
S23_D14 A-U	2.03	1.65	1.23	0.97	2.03	1.65	1.23	0.22
S23_D14 H-U	0.99	1.75	0.56	0.97	0.99	1.75	0.56	0.57
S23_D14 S-U	0.93	1.47	0.63	0.97	0.93	1.47	0.63	0.53
S23_D15 A-U	1.19	1.65	0.72	0.97	1.19	1.65	0.72	0.47
S23_D15 H-U	-0.28	1.75	-0.16	0.99	-0.28	1.75	-0.16	0.87
S23_D15 S-U	0.01	1.47	0.00	1.00	0.01	1.47	0.00	1.00
S23_D16 A-U	0.54	1.65	0.33	0.97	0.54	1.65	0.33	0.74
S23_D16 H-U	0.19	1.75	0.11	1.00	0.19	1.75	0.11	0.92
S23_D16 S-U	-0.24	1.47	-0.17	0.99	-0.24	1.47	-0.17	0.87
**S5_D3 A-U**	3.96	1.65	2.40	0.97	**3.96**	**1.65**	**2.40**	**0.02**
S5_D3 H-U	0.58	1.75	0.33	0.97	0.58	1.75	0.33	0.74
S5_D3 S-U	-0.48	1.47	-0.33	0.97	-0.48	1.47	-0.33	0.74
S6_D2 A-U	1.37	1.65	0.83	0.97	1.37	1.65	0.83	0.40
**S6_D2 H-U**	3.47	1.75	1.98	0.97	**3.47**	**1.75**	**1.98**	**0.05**
S6_D2 S-U	0.79	1.47	0.53	0.97	0.79	1.47	0.53	0.59

S6_D4 A-U	1.59	1.65	0.97	0.97	1.59	1.65	0.97	0.33
S6_D4 H-U	2.85	1.75	1.62	0.97	2.85	1.75	1.62	0.10
S6_D4 S-U	0.71	1.47	0.48	0.97	0.71	1.47	0.48	0.63
S18_D10 A-U	-0.35	1.65	-0.21	0.98	-0.35	1.65	-0.21	0.83
S18_D10 H-U	1.63	1.75	0.93	0.97	1.63	1.75	0.93	0.35
S18_D10 S-U	1.15	1.47	0.78	0.97	1.15	1.47	0.78	0.44
S19_D10 A-U	1.14	1.65	0.69	0.97	1.14	1.65	0.69	0.49
**S19_D10 H-U**	3.76	1.75	2.14	0.97	**3.76**	**1.75**	**2.14**	**0.03**
S19_D10 S-U	1.75	1.47	1.19	0.97	1.75	1.47	1.19	0.24
S10_D4 A-U	2.37	1.65	1.44	0.97	2.37	1.65	1.44	0.15
S10_D4 H-U	1.97	1.75	1.12	0.97	1.97	1.75	1.12	0.26
S10_D4 S-U	1.07	1.47	0.72	0.97	1.07	1.47	0.72	0.47
S20_D12 A-U	-0.05	1.65	-0.03	1.00	-0.05	1.65	-0.03	0.98
S20_D12 H-U	-1.63	1.75	-0.93	0.97	-1.63	1.75	-0.93	0.35
S20_D12 S-U	0.31	1.47	0.21	0.98	0.31	1.47	0.21	0.83

Table S3D. HbR: Contrast between each emotion and unemotional for channel in which speech evoked at least one significant haemodynamic response (HbO or HbR for any condition). Significance of contrasts, bold font indicates *p*<0.05.

		FDR corrected				Uncorrected		
**Contrast**	**Estimate**	**SE**	**z**	**p**	**Estimate**	**SE**	**z**	**p**
S7_D6 A-U	-0.22	0.76	-0.29	1.00	-0.22	0.76	-0.29	0.77
S7_D6 H-U	0.06	0.79	0.07	1.00	0.06	0.79	0.07	0.94
S7_D6 S-U	1.38	0.74	1.85	0.84	1.38	0.74	1.85	0.06
S9_D6 A-U	0.09	0.76	0.11	1.00	0.09	0.76	0.11	0.91
S9_D6 H-U	0.79	0.79	1.00	1.00	0.79	0.79	1.00	0.32
S9_D6 S-U	0.97	0.74	1.30	1.00	0.97	0.74	1.30	0.19
S9_D7 A-U	0.25	0.76	0.32	1.00	0.25	0.76	0.32	0.75
S9_D7 H-U	1.05	0.79	1.33	1.00	1.05	0.79	1.33	0.18
S9_D7 S-U	0.97	0.74	1.30	1.00	0.97	0.74	1.30	0.19
S10_D6 A-U	-0.27	0.76	-0.35	1.00	-0.27	0.76	-0.35	0.72
S10_D6 H-U	0.49	0.79	0.62	1.00	0.49	0.79	0.62	0.54
S10_D6 S-U	0.25	0.74	0.33	1.00	0.25	0.74	0.33	0.74
S11_D6 A-U	-0.30	0.76	-0.39	1.00	-0.30	0.76	-0.39	0.70
S11_D6 H-U	0.73	0.79	0.91	1.00	0.73	0.79	0.91	0.36
S11_D6 S-U	0.49	0.74	0.66	1.00	0.49	0.74	0.66	0.51
S11_D7 A-U	-0.48	0.76	-0.63	1.00	-0.48	0.76	-0.63	0.53
S11_D7 H-U	0.07	0.74	0.09	1.00	0.07	0.74	0.09	0.93
S11_D7 S-U	0.07	0.74	0.09	1.00	0.07	0.74	0.09	0.93
S11_D8 A-U	-0.70	0.76	-0.92	1.00	-0.70	0.76	-0.92	0.36
S11_D8 H-U	-0.08	0.79	-0.10	1.00	-0.08	0.79	-0.10	0.92
S11_D8 S-U	-0.58	0.74	-0.78	1.00	-0.58	0.74	-0.78	0.44
S19_D14 A-U	0.15	0.76	0.20	1.00	0.15	0.76	0.20	0.84
S19_D14 H-U	0.54	0.79	0.69	1.00	0.54	0.79	0.69	0.49
S19_D14 S-U	-0.05	0.74	-0.07	1.00	-0.05	0.74	-0.07	0.94
S21_D14 A-U	0.40	0.76	0.53	1.00	0.40	0.76	0.53	0.60
S21_D14 H-U	0.63	0.79	0.79	1.00	0.63	0.79	0.79	0.43
S21_D14 S-U	0.29	0.74	0.39	1.00	0.29	0.74	0.39	0.69
S21_D15 A-U	0.75	0.76	0.98	1.00	0.75	0.76	0.98	0.33
S21_D15 H-U	0.61	0.79	0.77	1.00	0.61	0.79	0.77	0.44
S21_D15 S-U	0.68	0.74	0.91	1.00	0.68	0.74	0.91	0.36
S22_D14 A-U	0.42	0.76	0.55	1.00	0.42	0.76	0.55	0.58
S22_D14 H-U	0.22	0.79	0.28	1.00	0.22	0.79	0.28	0.78
S22_D14 S-U	0.18	0.74	0.24	1.00	0.18	0.74	0.24	0.81
S22_D16 A-U	-0.10	0.76	-0.13	1.00	-0.10	0.76	-0.13	0.89

S22_D16 H-U	0.01	0.79	0.02	1.00	0.01	0.79	0.02	0.99
S22_D16 S-U	0.84	0.74	1.13	1.00	0.84	0.74	1.13	0.26
S23_D14 A-U	0.31	0.76	0.41	1.00	0.31	0.76	0.41	0.68
S23_D14 H-U	0.00	0.79	0.00	1.00	0.00	0.79	0.00	1.00
S23_D14 S-U	0.26	0.74	0.35	1.00	0.26	0.74	0.35	0.73
S23_D15 A-U	0.10	0.76	0.13	1.00	0.10	0.76	0.13	0.89
S23_D15 H-U	0.04	0.79	0.05	1.00	0.04	0.79	0.05	0.96
S23_D15 S-U	-0.16	0.74	-0.22	1.00	-0.16	0.74	-0.22	0.83
S23_D16 A-U	-0.19	0.76	-0.25	1.00	-0.19	0.76	-0.25	0.80
S23_D16 H-U	0.16	0.79	0.20	1.00	0.16	0.79	0.20	0.84
S23_D16 S-U	0.22	0.74	0.30	1.00	0.22	0.74	0.30	0.76
**S5_D3 A-U**	1.49	0.76	1.94	0.84	**1.49**	**0.76**	**1.94**	**0.05**
S5_D3 H-U	-0.02	0.79	-0.02	1.00	-0.02	0.79	-0.02	0.98
S5_D3 S-U	-0.25	0.74	-0.34	1.00	-0.25	0.74	-0.34	0.73
S6_D2 A-U	0.03	0.76	0.04	1.00	0.03	0.76	0.04	0.97
**S6_D2 H-U**	2.01	0.79	2.55	0.71	**2.01**	**0.79**	**2.55**	**0.01**
S6_D2 S-U	0.08	0.74	0.11	1.00	0.08	0.74	0.11	0.92
S6_D4 A-U	0.26	0.76	0.34	1.00	0.26	0.76	0.34	0.74
S6_D4 H-U	1.27	0.79	1.61	1.00	1.27	0.79	1.61	0.11
S6_D4 S-U	-0.12	0.74	-0.16	1.00	-0.12	0.74	-0.16	0.87
S18_D10 A-U	0.50	0.76	0.66	1.00	0.50	0.76	0.66	0.51
S18_D10 H-U	0.91	0.79	1.16	1.00	0.91	0.79	1.16	0.25
S18_D10 S-U	-0.05	0.74	-0.07	1.00	-0.05	0.74	-0.07	0.95
S19_D10 A-U	0.77	0.76	1.01	1.00	0.77	0.76	1.01	0.31
**S19_D10 H-U**	1.70	0.79	2.16	0.84	**1.70**	**0.79**	**2.16**	**0.03**
S19_D10 S-U	0.07	0.74	0.09	1.00	0.07	0.74	0.09	0.93
S10_D4 A-U	-0.01	0.76	-0.01	1.00	-0.01	0.76	-0.01	0.99
**S10_D4 H-U**	1.63	0.79	2.07	0.84	**1.63**	**0.79**	**2.07**	**0.04**
S10_D4 S-U	0.61	0.74	0.82	1.00	0.61	0.74	0.82	0.41
S20_D12 A-U	-0.81	0.76	-1.06	1.00	-0.81	0.76	-1.06	0.29
S20_D12 H-U	-0.37	0.79	-0.46	1.00	-0.37	0.79	-0.46	0.64
S20_D12 S-U	-0.22	0.74	-0.29	1.00	-0.22	0.74	-0.29	0.77

S4 – Supplementary materials for fNIRS session 2 (*happy* and sad in natural speech and speech with attenuated F0 cues)

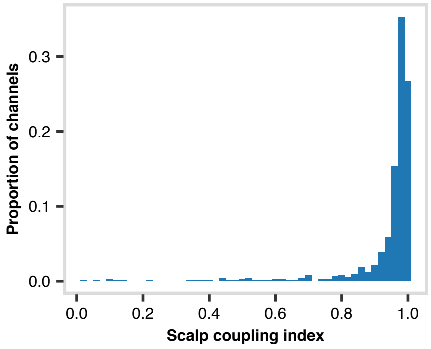

Figure S4A. Signal quality as measured by scalp-coupling index per channel for the second fNIRS session; histogram showing the distribution of scalp-coupling indices, calculated per channel, as a proportion of the total number of channels (N=2856).

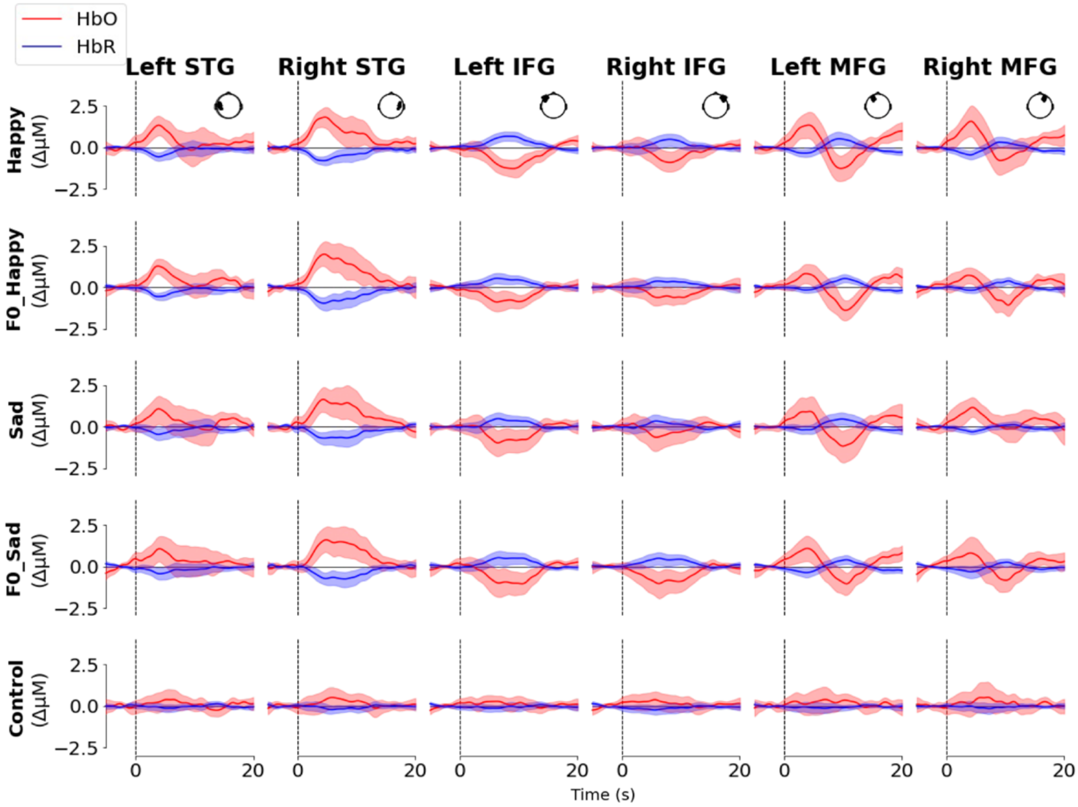

Figure S4B. Waveforms including MFG for session 2 (*happy* and *sad* in natural speech and speech with attenuated F0 variations).

**Model 9** *All emotions*	Accuracy ~ Condition + Test + (1+Condition‖Participant) + (1|Stimulus) + (1+Condition+Test‖Emotion)				
	$R^2_{m/c}$ =0.50/0.61				
	β	***SE***	***CI***	**z**	***p***
Intercept	3.59	0.71	1.91, 5.75	5.07	<0.001
Condition	-4.05	1.16	-7.09, -1.21	-5.54	<0.001
Test	0.49	0.36	-0.43, 1.43	1.35	0.178

Table S4A. Contrast coefficients for model predicting behavioural accuracy

		HbO					HbR				
		Model 10: *β ~ -1 + ROI + Condition + ROI:Condition + (1 + Condition|Participant)*					***Model 11:*** *β ~ -1 + ROI + Condition + ROI:Condition + (1 + Condition|Participant)*				
		$R^2_{m/c}$=0.16/0.55					$R^2_{m/c}$=0.18/0.53				
	**ROI**	**β**	***SE***	***CI***	***t***	***p***	**β**	***SE***	***CI***	***t***	***p***
**Happy**	LSTG	0.71	0.60	-0.50, 1.91	1.18	0.244	**-0.83**	**0.28**	**-1.38, -0.27**	**-2.97**	**0.004**
	RSTG	**1.42**	**0.60**	**0.22, 2.63**	**2.37**	**0.022**	**-1.37**	**0.28**	**-1.92, -0.81**	**-4.90**	**<0.001**
	LIFG	-0.65	0.60	-1.86, 0.56	-1.06	0.283	0.44	0.28	-0.12, 1.00	1.56	0.123
	RIFG	-0.40	0.60	-1.61, 0.81	-0.67	0.507	0.08	0.29	-0.49, 0.65	0.28	0.782
**Sad**	LSTG	**1.39**	**0.57**	**0.25, 2.54**	**2.43**	**0.018**	-0.51	0.29	-1.10, 0.06	-1.77	0.081
	RSTG	**2.57**	**0.58**	**1.41, 3.74**	**4.425**	**<0.001**	**-1.32**	**0.29**	**-1.89, -0.75**	**-4.61**	**<0.001**
	LIFG	-0.39	0.57	-1.54, 0.75	-0.69	0.493	0.53	0.29	-0.04, 1.10	1.85	0.069
	RIFG	0.16	0.57	-0.98, 1.31	0.28	0.778	0.12	0.29	-0.45, 0.68	0.40	0.688
**F0_happy**	LSTG	**2.11**	**0.60**	**0.91, 3.30**	**3.54**	**0.001**	**-0.67**	**0.33**	**-1.33, -0.02**	**-2.06**	**0.045**
	RSTG	**3.37**	**0.60**	**2.17, 4.56**	**5.66**	**<0.001**	**-1.15**	**0.33**	**-1.80, -0.50**	**-3.53**	**0.001**
	LIFG	0.10	0.60	-1.09, 1.29	0.17	0.868	0.41	0.33	-0.25, 1.06	1.25	0.216
	RIFG	0.71	0.60	-0.49, 1.90	1.19	0.241	0.12	0.33	-0.53, 0.78	0.38	0.705
**F0_sad**	LSTG	**1.32**	**0.62**	**0.66, 2.57**	**2.12**	**0.040**	**-1.21**	**0.34**	**-1.90, -0.52**	**-3.52**	**0.001**
	RSTG	**2.46**	**0.63**	**1.20, 3.73**	**3.90**	**<0.001**	**-1.93**	**0.34**	**-2.62, -1.23**	**-5.60**	**<0.001**
	LIFG	-1.05	0.62	-2.30, 0.20	-1.69	0.098	0.07	0.34	-0.63, 0.76	0.19	0.849
	RIFG	-0.47	0.62	-1.73, 0.78	-0.76	0.450	-0.12	0.34	-0.82, 0.57	-0.34	0.734
**Control**	LSTG	0.02	0.52	-1.02, 1.06	0.04	0.971	-0.12	0.31	-0.74, 0.49	-0.40	0.689
	RSTG	0.49	0.52	-0.55, 1.53	0.95	0.348	-0.24	0.31	-0.86, 0.38	-0.77	0.442
	LIFG	0.18	0.52	-0.86, 1.22	0.35	0.730	-0.31	0.31	-0.93, 0.31	-1.01	0.317
	RIFG	0.31	0.52	-0.73, 1.35	0.60	0.549	-0.31	0.31	-0.93, 0.31	-1.01	0.318

Table S4B. Group-level estimates of haemodynamic response amplitude per condition. Significance of contrasts, bold font indicates p<0.05.

	HbO					HbR				
	**Model 12:** β ~ -1 + ROI + Accuracy + ROI: Accuracy + (1 + Condition|Participant)					**Model 13:** β ~ -1 + ROI + Accuracy + ROI: Accuracy + (1 + Condition|Participant)				
	$R^2_{m/c}$=0.16/0.55					$R^2_{m/c}$=0.22/0.58				
**ROI**	**β**	***SE***	***CI***	***t***	***p***	**β**	***SE***	***CI***	***t***	***p***
LSTG	0.03	2.36	-4.73, 4.80	0.01	0.990	-1.14	1.16	-3.47, 1.20	-0.98	0.331
RSTG	**-5.33**	**2.36**	**-10.10, -0.55**	**-2.26**	**0.030**	**3.63**	**1.16**	**1.29, 5.96**	**3.14**	**0.003**
LIFG	-2.67	2.36	-7.44, 2.11	-1.13	0.265	-0.28	1.16	-2.62, 2.06	-0.24	0.811
RIFG	-3.33	2.36	-8.11, 1.44	-1.41	0.166	-0.05	1.16	-2.40, 2.30	-0.05	0.963

Table S4C. Relationship between speech-evoked haemodynamic activity and behavioural accuracy of emotion recognition in speech with uninformative F0 cues. Slope estimates, bold font indicates p<0.05. Importantly, likelihood ratio tests indicated that a *Condition:Accuracy or ROI:Condition:Accuracy* interaction did not explain substantially more variance, and as such models with these terms were not reported.

	Negatively correlated pairs					All pairs				
	**Model 14:** difference ~ ROI + Accuracy + ROI: Accuracy + (1 + Condition|Participant)					**Model 15:** difference ~ ROI + Accuracy + ROI: Accuracy + (1 + Condition|Participant)				
	$R^2_{m/c}$=0.34/0.78					$R^2_{m/c}$=0.30/0.61				
**ROI**	**β**	***SE***	***CI***	***t***	***p***	**β**	***SE***	***CI***	***t***	***p***
LSTG	3.12	3.08	-3.19, 9.44	1.02	0.319	1.16	2.56	-4.03, 6.34	0.45	0.653
RSTG	**-7.87**	**3.06**	**-14.15, -1.58**	**-2.57**	**0.016**	**-8.27**	**2.57**	**-13.46, -3.07**	**-3.22**	**0.003**
LIFG	-2.10	4.04	-10.17, 5.96	-0.52	0.604	-2.12	2.56	-7.30, 3.07	-0.83	0.413
RIFG	-6.15	3.76	-13.69, 1.38	-1.63	0.108	-2.85	2.56	-8.04, 2.33	-1.11	0.273

Table S4D. Relationship between haemodynamic response magnitude and behavioural accuracy of emotion recognition in speech with uninformative F0 cues. Slope estimates, bold font indicates p<0.05.

References

Al-Radhi, M. S., Csapó, T. G., & Németh, G. (2019). Adaptive refinements of pitch tracking and HNR estimation within a vocoder for statistical parametric speech synthesis. *Applied Sciences*, *9*(12), 2460. https://doi.org/10.3390/app9122460

Belin, P., Fillion-Bilodeau, S., & Gosselin, F. (2008). The Montreal Affective Voices: A validated set of nonverbal affect bursts for research on auditory affective processing. *Behavior Research Methods*, *40*(2), 531–539. https://doi.org/10.3758/BRM.40.2.531

Benjamini, Y., & Hochberg, Y. (1995). Controlling the false discovery rate: A practical and powerful approach to multiple testing. *Journal of the Royal Statistical Society: Series B (Methodological)*, *57*(1), 289–300. https://doi.org/10.1111/j.2517-6161.1995.tb02031.x

Bittner, R. M., Humphrey, E., & Bello, J. P. (2016). Pysox: Leveraging the Audio Signal Processing Power of Sox in Python. *17th International Society for Music In- Formation Retrieval Conference*, 4–6.

Boersma, P., & Weenink, D. (2018). Praat: Doing phonetics by computer. In *Version*. http://www.praat.org/

Cui, X., Bray, S., & Reiss, A. L. (2010). Functional near infrared spectroscopy (NIRS) signal improvement based on negative correlation between oxygenated and deoxygenated hemoglobin dynamics. *NeuroImage*, *49*(4), 3039–3046. https://doi.org/10.1016/j.neuroimage.2009.11.050

Delpy, D. T., Cope, M., Van Der Zee, P., Arridge, S., Wray, S., & Wyatt, J. (1988). Estimation of optical pathlength through tissue from direct time of flight measurement. *Physics in Medicine and Biology*, *33*(12), 1433–1442. https://doi.org/10.1088/0031-9155/33/12/008

Drugman, T., Huybrechts, G., Klimkov, V., & Moinet, A. (2018). Traditional machine learning for pitch detection. *IEEE Signal Processing Letters*, *25*(11), 1745–1749. https://doi.org/10.1109/LSP.2018.2874155

Fishburn, F. A., Ludlum, R. S., Vaidya, C. J., & Medvedev, A. V. (2019). Temporal Derivative Distribution Repair (TDDR): A motion correction method for fNIRS. *NeuroImage*, *184*, 171–179. https://doi.org/10.1016/j.neuroimage.2018.09.025

Gilbers, S., Fuller, C., Gilbers, D., Broersma, M., Goudbeek, M., Free, R., & Başkent, D. (2015). Normal-hearing listeners' and cochlear implant users' perception of pitch cues in emotional speech. *I-Perception*, *6*(5), 1–19. https://doi.org/10.1177/0301006615599139

Kocsis, L., Herman, P., & Eke, A. (2006). The modified Beer-Lambert law revisited. *Physics in Medicine and Biology*, *51*(5), N91–N98. https://doi.org/10.1088/0031-9155/51/5/N02

Luo, X., Fu, Q. J., & Galvin, J. J. (2007). Vocal emotion recognition by normal-hearing listeners and cochlear implant users. *Trends in Amplification*, *11*(4), 301–315. https://doi.org/10.1177/1084713807305301

Morise, M. (2015). CheapTrick, a spectral envelope estimator for high-quality speech synthesis. *Speech Communication*, *67*, 1–7. https://doi.org/10.1016/j.specom.2014.09.003

Morise, M. (2016). D4C, a band-aperiodicity estimator for high-quality speech synthesis. *Speech Communication*, *84*, 57–65. https://doi.org/10.1016/j.specom.2016.09.001

Morise, M., Yokomori, F., & Ozawa, K. (2016). WORLD: A vocoder-based high-quality speech synthesis system for real-time applications. *IEICE Transactions on Information and Systems*, *E99.D*(7), 1877–1884. https://doi.org/10.1587/transinf.2015EDP7457

Most, T., & Aviner, C. (2009). Auditory, visual, and auditory—Visual perception of emotions by individuals with cochlear implants, hearing aids, and normal hearing. *Journal of Deaf Studies and Deaf Education*, *14*(4), 449–464. https://doi.org/10.1093/deafed/enp007

Muges. (2017). *AudioTSM*. https://github.com/Muges/audiotsm/

Murray, I. R., & Arnott, J. L. (1993). Toward the simulation of emotion in synthetic speech: A review of the literature on human vocal emotion. *Journal of the Acoustical Society of America*, *93*(2), 1097–1108. https://doi.org/10.1121/1.405558

Paulmann, S., & Kotz, S. A. (2008). Early emotional prosody perception based on different speaker voices. *NeuroReport*, *19*(2), 209–213. https://doi.org/10.1097/WNR.0b013e3282f454db

Paulmann, S., Pell, M. D., & Kotz, S. A. (2008). How aging affects the recognition of emotional speech. *Brain and Language*, *104*(3), 262–269. https://doi.org/10.1016/j.bandl.2007.03.002

Paulmann, S., & Uskul, A. K. (2014). Cross-cultural emotional prosody recognition: Evidence from Chinese and British listeners. *Cognition and Emotion*, *28*(2), 230–244. https://doi.org/10.1080/02699931.2013.812033

Pell, M. D. (1998). Recognition of prosody following unilateral brain lesion: Influence of functional and structural attributes of prosodic contours. *Neuropsychologia*, *36*(8), 701–715. https://doi.org/10.1016/S0028-3932(98)00008-6

Pollermann, B. Z., & Archinard, M. (2002). Acoustic patterns of emotions. In & M. H. E. Keller. G. Bailly, A. Monaghan, J. Terken (Ed.), *Improvements in speech synthesis*. J. Wiley. https://doi.org/10.1002/0470845945.ch23

Pollonini, L., Olds, C., Abaya, H., Bortfeld, H., Beauchamp, M. S., & Oghalai, J. S. (2014). Auditory cortex activation to natural speech and simulated cochlear implant speech measured with functional near-infrared spectroscopy. *Hearing Research*, *309*, 84–93. https://doi.org/10.1016/j.heares.2013.11.007

R Core Team. (2020). *R: A language and environment for statistical computing*. R Foundation for Statistical Computing. http://www.r-project.org/

RStudio Team. (2020). *RStudio: Integrated Development for R*. RStudio, Inc. http://www.rstudio.com/

Saager, R. B., & Berger, A. J. (2005). Direct characterization and removal of interfering absorption trends in two-layer turbid media. *Journal of the Optical Society of America A*, *22*(9), 1874–1882. https://doi.org/10.1364/JOSAA.22.001874

Scherer, K. R., Banse, R., & Wallbott, H. G. (2001). Emotion inferences from vocal expression correlate across languages and cultures. *Journal of Cross-Cultural Psychology*, *32*(1), 76–92. https://doi.org/10.1177/0022022101032001009

Scholkmann, F., Klein, S. D., Gerber, U., Wolf, M., & Wolf, U. (2014). Cerebral hemodynamic and oxygenation changes induced by inner and heard speech: A study combining functional near-infrared spectroscopy and capnography. *Journal of Biomedical Optics*, *19*(1), 017002. https://doi.org/10.1117/1.jbo.19.1.017002

Verhelst, W., & Roelands, M. (1993). Overlap-add technique based on waveform similarity (WSOLA) for high quality time-scale modification of speech. *IEEE International Conference on Acoustics, Speech and Signal Processing*, *2*, 554–557. https://doi.org/10.1109/icassp.1993.319366

Vicenik, C. (n.d.-a). *Intensity-neutralizer*. http://phonetics.linguistics.ucla.edu/facilities/acoustic/praat.html

Vicenik, C. (n.d.-b). *Intensity-scaler*. http://phonetics.linguistics.ucla.edu/facilities/acoustic/praat.html

Weder, S., Zhou, X., Shoushtarian, M., Innes-brown, H., McKay, C., Olivares, V., Zhou, X., Innes-brown, H., & McKay, C. (2018). Cortical processing related to intensity of a modulated noise stimulus—A functional near-infrared study. *Journal of the Association for Research in Otolaryngology*, *19*(3), 273–286. https://doi.org/10.1007/s10162-018-0661-0

Yildirim, S., Bulut, M., Lee, C. M., Kazemzadeh, A., Busso, C., Deng, Z., Lee, S., & Narayanan, S. (2004). An acoustic study of emotions expressed in speech. *8th International Conference on Spoken Language Processing*, 2193–2196.
